# Modulation of marble-burying behavior in adult versus adolescent C57BL/6J mice of both sexes by ethologically relevant chemosensory stimuli

**DOI:** 10.1093/oons/kvae009

**Published:** 2024-05-24

**Authors:** C Leonardo Jimenez Chavez, Karen K Szumlinski

**Affiliations:** Department of Psychological and Brain Sciences, Psychology Building, U Cen Road, University of California Santa Barbara, Santa Barbara, CA 93106-9660, USA; Department of Psychological and Brain Sciences, Psychology Building, U Cen Road, University of California Santa Barbara, Santa Barbara, CA 93106-9660, USA; Department of Molecular, Cellular and Developmental Biology and the Neuroscience Research Institute, Bio II Building, U Cen Road, University of California Santa Barbara, Santa Barbara, CA 93106-9625, USA

**Keywords:** marble-burying test, sex differences, age-related differences, anxiety, chemosensory stimuli, adolescence, odor aversion

## Abstract

The marble-burying test is a pharmacologically validated paradigm used to study anxiety-like behaviors in laboratory rodents. Our laboratory has employed this assay as part of a behavioral screen to examine drug-induced negative affective states. Historically, the majority of our prior binge alcohol-drinking studies employed male subjects exclusively and reliably detected adolescent-adult differences in both basal and alcohol withdrawal-induced negative affect. However, age-related differences in marble-burying behavior were either absent or opposite those observed in our prior work when female subjects were included in the experimental design. As chemosensory cues from females are reported to be anxiolytic in males, the present study examined how odors from adult members of the opposite and same sex (obtained from soiled bedding) influence marble-burying behavior in adult, as well as adolescent, mice. Control studies examined the responsiveness of mice in the presence of novel neutral (vanilla) and aversive (tea tree) odors. Adult males exhibited reduced signs of anxiety-like behavior in the presence of female-soiled bedding, while adult females and adolescent mice of both sexes increased marble-burying behavior in the presence of both male- and female-soiled bedding. All mice exhibited increased burying in the presence of an aversive odor, while only adolescents increased marble-burying in response to the novel neutral odor. These data indicate sex by age interactions in the effects of volatile and nonvolatile odors from sexually-naive adult conspecifics on indices of anxiety-like behavior in the marble-burying test of relevance to the experimental design and procedural timing of experiments including sex as a biological variable.

## INTRODUCTION

Affective disorders exhibit a very high degree of comorbidity with alcohol use disorders [1], with the global prevalence of an affective disorder diagnosis being twice as high in women compared to men [2]. Concerningly, women advance through the addiction landscape at a more rapid rate than men [2–5] and exhibit higher rates of comorbid psychiatric conditions, including affective disorders [6–10]. The sexual dimorphism in alcohol use disorders, affective disorders and their comorbidity is theorized to appear early in adolescence [8, 11–13]; however, it is difficult to disentangle cause-effect relationships through studies of humans in an experimentally controlled fashion. Thus, we [14–17] and others, e.g. [18–21], have employed animal models to try to understand how biological sex interacts with the age of alcohol-drinking onset to impact brain and behavior. However, despite assaying for behavioral signs across a number of different paradigms, our recent large-scale efforts to identify sex differences in the age-selective effects of alcohol withdrawal on negative affect [15, 16] have failed to replicate our prior results derived from studies of a single-sex [22–28], with either no or inverse age-related differences observed for both basal and alcohol withdrawal-induced anxiety-like behavior. These failures to replicate our prior work challenged us to determine what procedural variables might have negatively impacted our ability to detect not only our basic finding that early alcohol withdrawal induces a negative affective state in adult mice [22–28], but age differences therein [23–28].

In this report, we examined the possibility that sex-related pheromones might modulate affective behavior expressed in one of the assays that reliably detected age differences in alcohol withdrawal-induced negative affect when mice of a single sex were examined [22–28] – the marble-burying test. In this paradigm, a shorter latency to begin marble-burying and a higher frequency of this behavior is generally interpreted as increased levels of anxiety-like behavior or heightened negative affect. Although some have questioned the specificity of marble-burying as an indicator of anxiety, c.f., [29], the marble-burying assay has high bidirectional, predictive, validity for anxiety-like behavior as burying is reduced by pretreatment with various anxiolytic [30–35] or antidepressant drugs [30–37] and increased by stimulation of the central noradrenergic system [38–40]. Similarly, in our hands, unpleasant or aversive psychophysiological conditions in humans, such as early alcohol withdrawal [41, 42], reliably augment marble-burying behavior in adult male C57BL/6J (**B6**) mice [22–27], with a similar effect reported for B6 females [28]. In contrast, but consistent with the human condition [c.f. [43]], adolescent B6 mice are resilient to this anxiogenic/dysphoric state in early alcohol withdrawal, but manifest increased marble-burying behavior later in adulthood [e.g. [23–28]]. As exposure to female-related pheromones are reported to lower fear responses and induce greater risk taking in adult male mice [44–46], we hypothesized that our failure to replicate age-related differences in both basal and alcohol withdrawal-induced negative affect, particularly in male mice, might relate to the inadvertent exposure to female pheromones during testing. Further, as the behavioral response to sex-related pheromones can vary depending on the animal’s biological sex, reproductive physiology and experience [44, 47–49], the possibility existed that exposure to odors of the opposite sex may have also impacted our ability to detect age-related differences in marble-burying during alcohol withdrawal when both sexes were examined concurrently.

To the best of our knowledge, no study has explicitly examined how exposure to odors of the same or opposite sex impacts behavior in the marble-burying test, let alone how effects might vary as a function of biological sex or sexual maturity. Thus, the present study was designed to examine how sex-related pheromones alter marble-burying behavior in sex- and drug-naive adult and adolescent mice of both sexes. To facilitate interpretation of our results, follow-up studies examined for sex by age interactions in the effects of a novel neutral odor (vanilla) [50, 51] versus a novel, aversive odor (tea tree) [40] on marble-burying. Our results replicate the anxiogenic effect of tea tree odor on marble-burying [40], but also provide new evidence for clear sex- and age-dependent effects of exposure to male and female pheromones, as well as a neutral vanilla odor, on marble-burying behavior. These findings are of significant relevance to the experimental design and procedural timing of experiments including the subject factors of sex and age, particularly when comparing between adult and adolescent subjects. For transparency, the details of peer-review for this report are provided in the Supplementary Data.

## MATERIALS AND METHODS

### Subjects

The subjects of this study included male and female adult (PND 56+) and adolescent (PND 28–29) C57BL/6J mice (see Experimental Designs for more details). All mice were housed in same-sex, age-matched, groups of 2–4 per cage that were situated on ventilated racks. These polycarbonate cages contained sawdust bedding, nesting materials and an enrichment device and were located in a colony room with regulated climate and humidity. All mice were acclimated to a 12-h reversed light cycle, with lights turning off at 11:00 AM, in accordance with vivarium guidelines. To ensure minimal stress during testing, a minimum 7-day acclimation period was implemented, during which mice experienced limited handling restricted to tail-marking procedures. All experimental procedures were in compliance with The Guide for the Care and Use of Laboratory Animals (National Research Council, 2014) and approved by the Institutional Animal Care and Use Committee of the University of California, Santa Barbara.

### Marble-burying procedures

In preparation for behavioral testing, mice were relocated from the colony room to a designated testing room within the vivarium, where they remained in their home cages for a 30-min acclimation period prior to testing. For all experiments, a sex- and age-specific testing schedule was implemented to nullify the influence of subject-related odors from the opposite sex or differentially aged mice (i.e. odors not associated with the different bedding conditions employed). Mice were then subjected to the marble-burying assay over a span of two or three consecutive days, during which they encountered a different bedding condition each day. With some exceptions, detailed in the Experimental Designs section below, the sequence of bedding conditions was counterbalanced to mitigate order effects.

For testing, each mouse was introduced to a polycarbonate testing box (12 cm × 8 cm × 6 cm), which contained 5 cm of sawdust bedding (P.J Murphy Forest Products, Montville, NJ, USA) from one of the different bedding conditions (see Bedding Collection/Preparation below) and 20 uniformly distributed, round black glass marbles, arranged in a 4×5 grid. For a 20-min testing period, mice were free to explore and bury marbles where a more burying behavior (assayed by number of marbles buried at the end of the session and/or total time spent burying) greater frequency of burying behavior and a shorter latency to begin burying marbles served as indices of heightened negative affect. After each testing session, the total number of marbles covered 2/3rds by the bedding was documented, and the mice were returned to their home cages. Test boxes were completely emptied of bedding, sprayed thoroughly with a virucidal spray (Rescue™; Virox Technologies Inc., Oakville, ON, Canada) and then wiped dry, prior to the addition of new bedding for the next animal. Upon the completion of testing at the end of the day, all testing cages were sent for washing and cleaned cages were employed the next day. For Experiments 1 and 2, behavior was digitally video-recorded using AnyMaze tracking software (Stoeling Co., Wood Dale, IL, USA) for playback and determination of distance traveled (in m). The latency to start burying and/or the total time spent burying were then recorded by a blind observer using a stopwatch upon video playback, as conducted in previous studies, e.g. [14, 15]. For Experiment 3, a computer malfunction required that we score behavior manually as conducted in a recent study by our group [16] and thus, the data for the total time spent burying and the distance traveled could not be determined for this study.

### Bedding collection/preparation

For Experiment 1, we collected bedding that had been soiled over a six-day period from the home cages of adult, sex- and drug-naïve, B6 female and male mice 24 h prior to testing for marble-burying. The soiled bedding was always derived from cages of unfamiliar, adult, mice. To preserve the distinct scent profiles, the soiled bedding was collected and stored separately in sealed containers for each sex. On the test day, a 5 cm bedding mix was used, consisting of equal parts of soiled and unscented bedding for each mouse. For Experiments 2 and 3, the scented bedding (vanilla or tea tree) was freshly prepared on the day of testing. Clean, unscented, bedding was infused with artificial vanilla extract (McCormick & Co., Inc., Hunt Valley, MD, USA) or tea tree oil (rareESSENCE, LLC; Minneapolis, MN, USA) by sprinkling 5–6 drops onto the bedding in each apparatus and then thoroughly mixed to ensure uniform distribution of scent. To augment the sensory environment, an approximately 5 cm stripe of the vanilla extract or the tea tree oil was applied around the interior of the polycarbonate cage with a moistened Kimwipe (Kimberly-Clark, Irving, TX, USA), as conducted in prior studies of predator odor exposure conducted by our group [52, 53].

### Experimental designs

**Figure 1:**
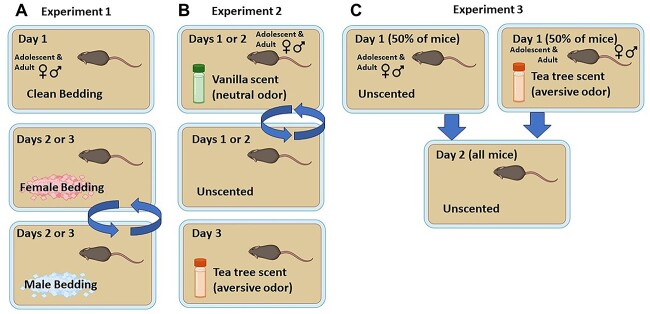
Summary of the experimental designs and procedural time-lines for (A) Experiment 1, (B) Experiment 2 and (C) Experiment 3 described in this report. All images were created in BioRender (biorender.com)

The primary objective of this study was to examine how ethologically relevant chemosensory stimuli within the marble-burying test modulate the burying behavior of both adolescent and adult, male and female, sex-naïve B6 mice. This study included three distinct, yet interrelated, experiments as detailed below. The experimental designs of the three experiments are graphically depicted in Fig. 1.

#### Experiment 1

The first experiment was designed to assay the influence of socially relevant chemosensory stimuli (odors from male and female mice) on three indices of affect in the marble-burying test: latency to first bury to gauge initial behavioral responsiveness to the marbles, as well as the time spent burying and total number of marbles buried as indices of negative affect, e.g. [36]. As the perception of odors from the opposite sex change during sexual development, e.g. [47], this study compared the marble-burying behavior of male and female adolescent, as well as male and female adult, mice in the presence of unscented bedding (i.e. standard procedures; control condition; Day 1 for all mice), or unscented bedding mixed with soiled bedding from cages of unfamiliar adult male or female mice (respectively, male- and female-soiled; see Fig. 1). The order of testing under the soiled bedding conditions was counterbalanced across the next two consecutive days within each age and sex. To minimize the influence of sex-related pheromones of the subjects, males and females were tested on distinct days and adolescents were tested separately from adults. Experiment 1 employed 48 mice (n = 12/sex/age) and all mice were obtained from The Jackson Laboratory (Sacramento, CA) and allowed to acclimate to the reverse cycle for at least 1 week prior to behavioral testing.

#### Experiment 2

Experiment 2 was designed to facilitate the interpretation of the effects of inherently motivating sex-specific chemosensory stimuli on marble burying behavior (i.e. do increased indices of marble-burying in the presence of a sex-related odor reflect a response to an aversive/anxiogenic stimulus or a mere response to a novel odor?). Specifically, we compared the marble-burying behavior of male and female mice, both adolescent and adult, in the presence of a novel but neutral, odor (artificial vanilla extract) [50, 51] against a novel, but inherently aversive, odor (tea tree oil) [40]. Experiment 2 also employed a within-subjects design in which mice were exposed to all three bedding conditions. However, out of concern that the tea tree odor might induce a conditioned place-aversion that could impact behavior in the presence of the unscented and vanilla-scented conditions [54], we counterbalanced the presentation of unscented and vanilla odor over the first two days of testing and then all mice were tested in the presence of the tea tree odor on the third and final day (see Fig. 1B). As in Experiment 1, a total of 48 mice were employed in Experiment 2 (n = 12/sex/age). However, due to pandemic-related difficulties acquiring adult mice, the adult mice in this study were obtained from our breeding colony at UCSB (breeders obtained originally from The Jackson Laboratory; offspring tested PNDs 60–75), while the adolescent mice were obtained directly from The Jackson Laboratory as in Experiment 1.

#### Experiment 3

The results of Experiment 2 confirmed that tea tree odor increased marble-burying behavior. Thus, we deemed it important for future work to determine whether initial exposure to tea tree oil would induce a conditioned aversive response to the marble-burying apparatus that might impact subsequent marble responsiveness. Although published studies indicated that marble-burying behavior under standard testing conditions neither habituates nor sensitizes with repeated testing in mice, e.g. [29], we first conducted a pilot study in adult male B6 mice from our breeding colony (aged 6–9 months) in which mice were tested in the presence of unscented bedding over the course of two consecutive days. Having established that the marble-burying behavior of mice from our breeding colony is also stable across days, we examined for carry-over effects of the tea tree odor in male and female, adolescent (PND 27–28) and adult mice (PND 60–75). Unfortunately, a computer malfunction around the time of testing precluded video-recording of behavior for playback to determine the latency to bury, the total time spent burying and the total distance traveled. Thus, only the data for the number of marbles buried is reported for Experiment 3. Consistent with Experiment 2, the adult mice were obtained from our breeding colony and the adolescent mice were obtained from The Jackson Laboratory. Experiment 3 employed a mixed design in which half of the mice were exposed to the unscented bedding across two consecutive days, while the other half of the mice were initially exposed to tea tree-scented bedding, followed by unscented bedding to examine for carry-over effects (see Fig. 1C). This study employed n = 7 adult mice/sex and n = 8 adolescent mice/sex.

### Statistical analyses and graphical depiction of findings

The data were analyzed using mixed-model ANOVAs, with Age and Sex as between-subjects variables and Bedding as the repeated measure variable for all analyses. Both the F statistics and effects sizes are reported. Significant main effects in the absence of any significant interaction(s) were followed by LSD post-hoc tests. Significant interactions were deconstructed along the relevant factors, followed by tests for simple effects with LSD corrections (when >2 comparisons were required) or paired samples t-tests (when 2 comparisons were required). Although the LSD method of post-hoc analyses is less conservative than other corrections (e.g. Tukey’s tests), it offers greater statistical power to detect group differences, which aligns with the exploratory nature of our study to discover potential factors that may modulate behavior. For all analyses, alpha was set at 0.05. When the assumption of sphericity was not met, analyses were corrected using the Greenhouse–Geisser adjustment. Extreme outliers, defined by ±3 × IQR, were removed. With this exclusion criterion, 1 adolescent female mouse was removed from the analysis of the latency to bury from Experiment 1, 1 adolescent female, 2 adolescent males and 1 adult female were removed from the analysis of the latency to bury from Experiment 2. No other animals were noted as extreme outliers. IBM SPSS Statistics software (version 27.0 for Macintosh) was used for all statistical tests. For transparency, the results for adult male, adult female, adolescent male and adolescent female mice across the specific bedding conditions employed in each experiment are always presented, regardless of the statistical outcome for the specific analysis. Graphs summarizing significant interactions between our factors are also provided to facilitate visualization of the statistical results. When no interactions are observed between factors, significant main effects are also provided. GraphPad Prism software (version 10 for Macintosh) was used to create all graphs.

## RESULTS

### Experiment 1: sex by age interactions in the effects of social chemosensory stimuli on marble-burying behavior

To test the hypothesis that the apparent anxiolytic effect of female odors reported in prior studies of adult male mice [44–46] extends to marble-burying procedures, we employed a within-subjects design in which we compared marble-burying behavior in the presence of unscented bedding on day 1 of testing, followed by exposure to female- or male-soiled bedding in a counter-balanced fashion across days 2 and 3 of testing (see Fig. 1A). To examine whether the effects of social chemosensory stimuli were specific to adult males, the study also included adult females, as well as both female and male adolescent mice. Based on the limited literature e.g. [47], it was predicted that adolescent mice of both sexes, as well as adult females, would exhibit anxiety-like behavior as manifested by increased marble-burying in the presence of the bedding of adult males.

#### Latency to bury

**Figure 2:**
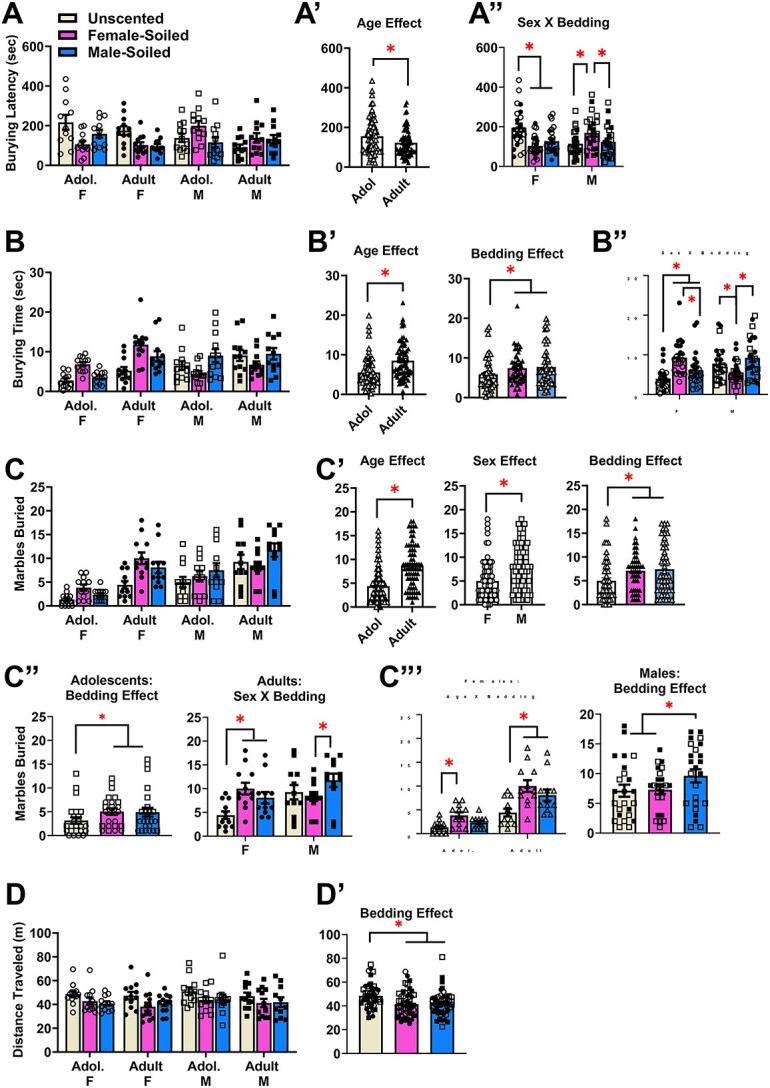
Summary of the effects of social chemosensory stimuli on the marble-burying behavior of adolescent and adult mice (Experiment 1). (**A**) Summary of the latency to first begin marble-burying exhibited by the adolescent (Adol.) and adult male (M) and female (F) mice in the presence of unscented, female- and male-soiled bedding for which a significant Sex by Bedding Condition interaction was detected [females: Adol. unscented (*n* = 11), female-soiled (*n* = 11), male-soiled (*n* = 12); for all other groups (*n’s* = 12)]. (**A**’) Depiction of the significant Age effect for the latency to bury. (**A**”) Depiction of the significant Sex × Bedding interaction for the latency to bury. (**B**) Results for the total time spent burying marbles indicated a significant Sex by Bedding Condition interaction [all group *n’s* = 12]. (**B**’) Depiction of the significant main effects of Age and Bedding Condition for the time spent burying. (**B**”) Depiction of the significant Sex × Bedding Condition interaction for the time spent burying. (**C**) Summary of the number of number of marbles buried by the mice in Experiment 1. (**C**’) Depiction of the significant Age, Sex and Bedding Condition effects for the number of marbles buried. (**C**”) Deconstruction of the significant 3-way interaction along the Age factor indicated a significant main Bedding Condition effect for adolescent mice and a significant Sex × Bedding Condition interaction for adults. (**C**‴) Deconstruction of the significant 3-way interaction along the Sex factor revealed a significant Age × Bedding interaction for female mice and a significant Bedding Condition effect for males. (**D**) Summary of distance traveled (in m) during Experiment 1 [adult males: unscented (*n* = 12), female-soiled (*n* = 12), male-soiled (*n* = 11); all other group *n’s* = 12]. (**D**’) Depiction of the significant main Bedding Condition effect for the distance traveled. The bar graphs represent the means ± SEMs of the number of individual mice indicated above. *P < 0.05 for indicated comparisons (LSD post-hoc tests)

A summary of the group differences in the latency to bury by the mice in Experiment 1 are presented in Fig. 2A. A mixed-model ANOVA was conducted to investigate the effect of Age (adolescent vs. adult), Sex (female vs. male), and Bedding Condition (unscented, female-soiled, and male-soiled) on the latency to first exhibit marble burying. The analysis yielded a significant main effect of Age (Adolescents > Adults; Fig. 2A’) [F(1,43) = 5.58, *P* = 0.023, eta = 0.115]. In contrast, the main effects of Sex [F(1,43) = 0.19, *P* = 0.664, eta = 0.004] and Bedding Condition [F(2,86) = 2.22, *P* = 0.115, eta = 0.049] were not statistically significant.

Interaction effects were also examined and although the ANOVA failed to detect a significant three-way interaction [3-way interaction: F(2,86) = 2.737, *P* = 0.070, eta = 0.060], a significant two-way interaction between Sex × Bedding Condition was found [F(2,86) = 12.973, *P* < 0.001, eta = 0.232]. Thus, the data were collapsed across Age and analyzed separately for male and female mice to determine how the soiled bedding influenced their initial response to the marbles. As depicted in Fig. 2A”, the latency of females to bury marbles was significantly shorter in female-soiled (*P* < 0.001) and male-soiled bedding (*P* = 0.002) compared to the unscented bedding condition, with no significant differences observed between the female- and male-soiled bedding (*P* = 0.269). As also illustrated in Fig. 2A” and in contrast to females, male mice exhibited a longer latency to bury when tested in female-soiled bedding, relative to both the unscented (*P* = 0.011) and male-soiled conditions (*P* = 0.026), with no effect of male-soiled bedding detected (*P* = 0.643). Thus, irrespective of age, these data for the latency to first marble bury indicate that female odors exert an anxiolytic effect in male mice, social chemosensory stimuli from both sexes are anxiogenic in females.

#### Total time spent burying

A summary of the group differences in the total time spent burying by the mice in Experiment 1 are presented in Fig. 2B. An Age × Sex × Bedding Condition mixed-model ANOVA was employed to examine for group differences in the total time spent marble burying. Results indicated significant main effects of Age (Adults > Adolescents; Fig. 2B’, left) [F(1,44) = 11.05, *P* = 0.002, eta = 0.201] and Bedding Condition (Female-soiled vs. Unscented, *P* = 0.007; Male-soiled vs Unscented, *P* = 0.002; Female-soiled vs. Male-soiled, *P* = 0.574; Fig. 2B’, right) [F(2,88) = 6.95, *P* = 0.002, eta = 0.136], but not of Sex [F(1,44) = 1.35, *P* = 0.252, eta = 0.030]. While the 3-way interaction was not statistically significant [3-way interaction: F(2,88) = 0.562, *P* = 0.572, eta = 0.013], a significant Sex × Bedding Condition was observed [F(2,88) = 30.984, *P* < 0.001, eta = 0.413] that is depicted in Fig. 2B”. As illustrated, simple main effects analysis with LSD post-hoc corrections revealed that female mice spent a longer time burying marbles in both the female-soiled (*P* < 0.001) and male-soiled bedding (*P* = 0.007), compared to the unscented bedding condition**.** Moreover, females spent more time marble burying in the female- versus male-soiled bedding (*P* < 0.001). In contrast, male mice spent less time burying marbles in the female-soiled bedding than in the unscented (*P* = 0.007) and male-soiled bedding (*P* < 0.001), with no significant difference detected between the unscented and male-soiled bedding conditions (Fig. 2B”; *P* = 0.062). These results further the notion that female odors exert an anxiolytic effect in male mice, while both male and female odors are anxiogenic in female mice.

#### Number of marbles buried

A summary of the group differences in the number of marbles buried by the mice in Experiment 1 are presented in Fig. 2C. We also conducted an Age × Sex × Bedding Condition mixed-model ANOVA to evaluate the total number of marbles buried during each testing session. Significant main effects were observed for Age (Adults > Adolescents; Fig. 2C’, left) [F(1,44) = 21.91, *P* < 0.001, eta = 0.332], Sex (Males > Females; Fig. 2C’, middle) [F(1,44) = 10.95, *P* = 0.002, eta = 0.199] and for Bedding Condition (Male- and Female-Soiled > Neutral, *P*’s < 0.001; Fig. 2C’, right) [F(2,88) = 13.49, *P* < 0.001, eta = 0.235].

Furthermore, a significant three-way interaction (Age × Sex × Bedding Condition) was detected [F(2,88) = 3.363, *P* = 0.039, eta = 0.071], indicating that the effects of Bedding Condition on marble-burying behavior varied as a function of both Age and Sex. To investigate further, the ANOVA was first deconstructed along the Age factor to examine the Sex by Bedding Condition interaction separately for adolescents and adults. For the adolescent mice, the Sex × Bedding interaction was not significant [F(2,44) = 2.582, *P* = 0.087, eta = 0.105], however, a main effect of Bedding Condition was detected for adolescent mice [F(2,44) = 5.987, *P* = 0.005, eta = 0.214], which is depicted in Fig. 2C” (left). As illustrated, LSD post-hoc tests indicated that adolescent mice buried more marbles in both the female-soiled (*P* = 0.004) and male-soiled bedding (*P* = 0.003), compared to the unscented bedding, with no significant differences between the male- versus female- soiled bedding conditions (*P* = 0.860). For the adults, a significant Sex × Bedding Condition interaction was observed [F(2,44) = 9.134, *P* < 0.001, eta = 0.293], which is depicted in Fig. 2C” (right). As illustrated, test for simple main effects with LSD corrections indicated that adult female mice buried more marbles in the presence of the female-soiled (*P* < 0.001) or male-soiled bedding conditions (*P* = 0.008), relative to unscented bedding, with no difference detected between the female-soiled and male-soiled conditions (*P* = 0.094). In contrast, adult male mice buried more marbles in male-soiled bedding compared to female-soiled bedding (*P* = 0.005); however, the number of marbles buried did not differ significantly between the unscented condition and the female- soiled (*P* = 0.402) or male-soiled bedding conditions (*P* = 0.061).

The significant 3-way ANOVA was then deconstructed along the Sex factor to explore age-related differences within each bedding environment within each sex. For females, the Age × Bedding Condition interaction was significant [F(2,44) = 5.078, *P* = 0.010, eta = 0.188]. As illustrated in Fig. 2C‴ (left), subsequent analysis for simple main effects with LSD post-hoc corrections revealed that adolescent females buried more marbles in the presence of female-soiled bedding than unscented bedding (*P* = 0.001), with no other significant group differences detected (unscented vs. male-soiled, *P* = 0.160; female vs. male-soiled; *P* = 0.094). In contrast, adult females buried more marbles in both the female-soiled (*P* < 0.001) and male-soiled environments (*P* < 0.001), compared to the unscented bedding. Moreover, female buried more marbles in the female- vs. the male-soiled bedding (*P* = 0.036; see Fig. 2C‴, left). For males, the Age × Bedding ANOVA failed to indicate a significant 2-way interaction [F(2,44) = 1.045, *P* = 0.359, eta = 0.045], but a significant overall main effect of Bedding Condition was observed that is illustrated [F(2,44) = 5.217, *P* = 0.009, eta = 0.192]. As shown in Fig. 2C‴ (right), LSD post-hoc tests indicated that this main effect reflected more marble burying in the male-soiled bedding, than both the female-soiled (*P* = 0.012) and unscented bedding conditions (*P* = 0.013), with no significant difference in the number of marbles buried between the unscented vs. female-soiled bedding environments (*P* = 0.824). These data for the number of marbles buried suggest that this variable may be most sensitive to age-related differences in the effects of socially relevant odors on behavior in this assay, revealing an anxiogenic effect of both male- and female odors.

#### Distance traveled

Analysis of the distance traveled by the mice during the 20-min marble-burying session (Fig. 2D) did not detect significant main effects for Age [F(1,43) = 1.08, *P* = 0.305, eta = 0.024] or Sex [F(1,43) = 0.43, *P* = 0.783, eta = 0.010]. However, a significant main effect of Bedding Condition was observed (Unscented > Female- and Male-soiled; *P*’s < 0.001) that is depicted in Fig. 2D’ [F(2,86) = 12.982, *P* < 0.001, eta = 0.232]. The analysis also revealed comparable locomotor activity between the female-soiled and male-soiled bedding conditions was comparable (*P* = 0.620). This analysis detected no significant Age × Sex × Bedding Condition interaction [F(2,86) = 0.221, *P* = 0.802, eta = 0.005], no significant Sex × Bedding Condition [F(2,86) = 0.072, *P* = 0.931, eta = 0.002] or Age × Bedding Condition interactions [F(2,86) = 0.451, *P* = 0.638, eta = 0.010]. These data for the distance travelled indicates that the presence of male or female odors lowers locomotor activity, regardless of the age or sex of the mice tested. Thus, an inverse relationship exists between the effects of socially relevant odors on psychomotor activation and our indices of anxiety-like behaviors in this assay, which may reflect the fact that marble-burying is physically incompatible with forward locomotion (i.e. animal is stationary over a marble when burying).

### Experiment 2: sex by age interactions in the effects of neutral versus aversive chemosensory stimuli on marble-burying behavior

To facilitate the interpretation of the results from Experiment 1, we conducted a second experiment in a distinct cohort of adult and adolescent, male and female, mice to examine marble-burying in the presence of a novel neutral versus novel aversive/anxiogenic odor (vanilla and tea tree, respectively). Based on the extant literature [40], it was predicted that tea tree odor would increase marble-burying in all mice, which would suggest that the increased marble-burying observed in response to sex-related odors in Experiment 1 reflects an aversive response/anxiety-like behavior. However, if a presumably neutral odor such as vanilla [50, 51] also increased marble-burying, then the increased marble-burying in response to sex-related odors in Experiment 1 might also reflect a mere response to a novel smell. The experimental design is summarized in Fig. 1B, with testing in the presence of the tea tree odor conducted on the third day out of concern that mice might develop a conditioned place-aversion that could negatively impact behavior.

#### Latency to start burying

**Figure 3:**
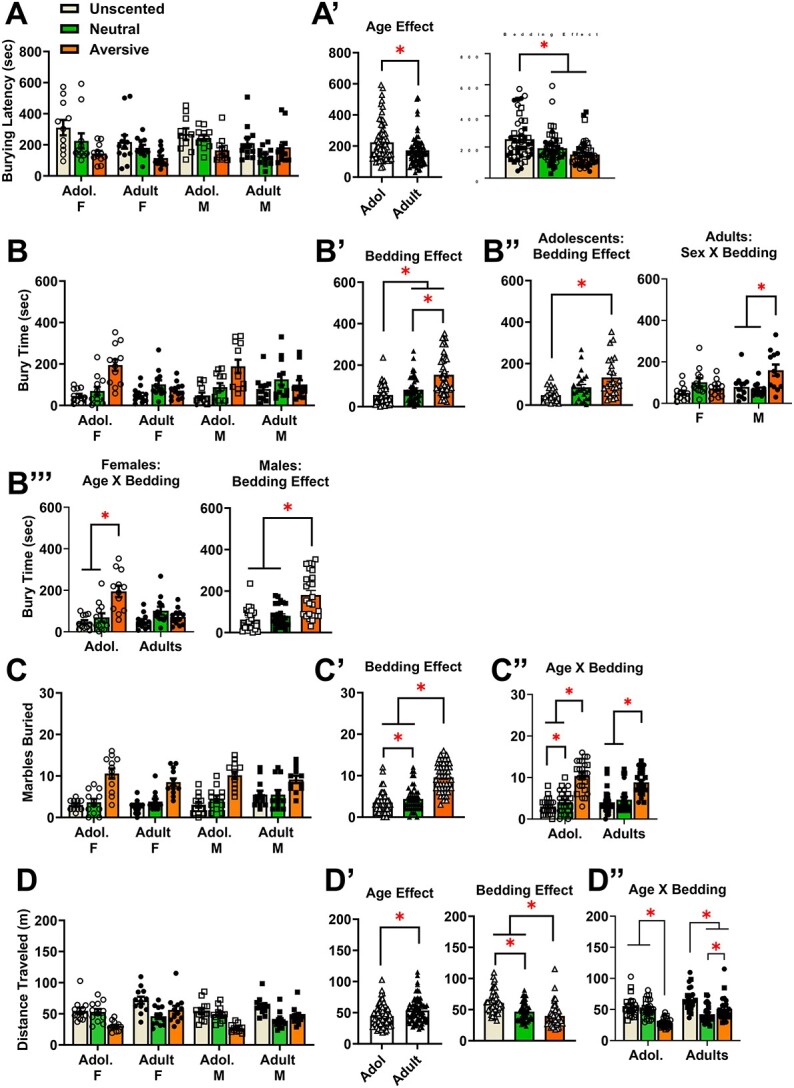
Summary of the effects of novel chemosensory stimuli on the marble-burying behavior of adolescent and adult mice (Experiment 2). (**A**) Summary of the latency to first begin marble-burying exhibited by the adolescent (Adol.) and adult male (M) and female (F) mice in the presence of unscented bedding or bedding scented with a neutral vanilla odor (Neutral) or a noxious tea tree odor (Aversive), for which a main Bedding effect was detected [females: Adol. unscented (*n* = 11), neutral-scented (*n* = 11), aversive-scented (*n* = 12); Adults unscented (*n* = 12), neutral-scented (*n* = 11), aversive-scented (*n* = 12); males: Adol. unscented (*n* = 10), neutral-scented (*n* = 11), aversive-scented (*n* = 12); Adults unscented (*n* = 12), neutral-scented (*n* = 12), aversive-scented (*n* = 12)]. (**A**’) Depiction of the significant Age and Bedding Condition effects for the latency to bury. (**B**) Summary of the time spent burying by the mice in Experiment 2. (**B**’) Depiction of the significant main Bedding Condition effect for the time spent burying. (**B**”) Deconstruction of the significant 3-way interaction for the time spent burying along the Age factor revealed a significant Bedding Condition effect for adolescent mice and a significant Sex × Bedding interaction for adults. (**B**‴) Deconstruction of the significant 3-way interaction along the Sex factor revealed a significant Age × Bedding interaction for female mice and a main Bedding Condition effect for males. (**C**) Summary of the number of marbles buried by the mice in Experiment. (**C**’) Depiction of the main Bedding Condition effect for the number of marbles buried. (**C**”) Depiction of the significant Age × Bedding interaction for this same variable. (**D**) Summary of the total distance traveled (in m) during Experiment 2. (**D**’) Depiction of the significant main effects of Age and Bedding condition for the distance traveled. (**D**”) Depiction of the significant Age × Bedding interaction for the distance traveled. The bar graphs represent the means ± SEMs of the number of individual mice indicated above. *P < 0.05 for indicated comparisons (LSD post-hoc tests)

A summary of the group differences in the latency to bury by the mice in Experiment 2 are presented in Fig. 3A. In examining the factors influencing the latency to initiate marble-burying, we ran a mixed-model ANOVA with Age (adolescent vs. adult) and Sex (female vs. male) as between-subject factors, and Bedding Condition (unscented, neutral/vanilla, and aversive/tea tree) as a within-subjects factor. The results indicated no significant main effect of Sex [F(1,38) = 0.028, *P* = 0.869, eta = 0.001]. Furthermore, the Age × Sex × Bedding Condition interaction was also non-significant [F(2,76) = 0.653, *P* = 0.523, eta = 0.017]. No other significant interactions were observed [Sex × Bedding ANOVA: F(2,76) = 1.756, *P* = 0.180, eta = 0.044; Age × Bedding ANOVA: F(2,76) = 1.395, *P* = 0.254, eta = 0.035].

We did however detect a significant main effect of Age [F(1,38) = 5.48, *P* = 0.025, eta = 0.126], that reflected a shorter latency to start burying marbles in the adult versus adolescent mice (Fig. 3A’, left). The analysis also detected a significant main Bedding Condition effect on the latency to start burying (Fig. 3A’, right) [F(2,76) = 8.552, *P* < 0.001, eta = 0.184]. As illustrated, LSD post-hoc tests indicated that this effect reflected a shorter latency to bury marbles in both the neutral vanilla (*P* = 0.030) and aversive tea tree scented bedding conditions (*P* < 0.001), relative to the unscented condition. Additionally, no significant overall difference was detected between the vanilla and tea tree-scented bedding (*P* = 0.066). These data for the latency to bury indicate that while adult mice are quicker to respond to the marbles than adolescent mice, irrespective of the odor present, novel odors of both neutral and aversive valence instigate marble-burying faster than unscented marbles, irrespective of the age or sex of the mouse.

#### Total time spent burying

A summary of the group differences in the total time spent burying by the mice in Experiment 2 are presented in Fig. 3B. An Age × Sex × Bedding Condition (unscented, neutral/vanilla, and aversive/tea tree) mixed-model ANOVA was employed to examine for group differences in the total time spent marble burying. The results indicated no significant main effects of Sex [F(1,44) = 1.423, *P* = 0.239, eta = 0.031] or Age [F(1,44) = 1.757, *P* = 0.192, eta = 0.038] or interaction between these variables [F(1,44) = 0.678, *P* = 0.415, eta = 0.015]. A significant Bedding Condition effect [unscented < vanilla and tea tree (*P*’s < 0.001); vanilla < tea tree (*P* = 0.035); F(2,88) = 34.448, *P* < 0.001, eta = 0.439] (Fig. 3B’) as was the 3-way interaction [F(1,88) = 4.613, *P* = 0.013, eta = 0.095]. To investigate further, the ANOVA was first deconstructed along the Age factor to examine the Sex by Bedding Condition interaction separately for adolescents and adults. For the adolescent mice, the Sex × Bedding interaction was not significant [F(2,44) = 0.215, *P* = 0.807, eta = 0.010], however, a main effect of Bedding Condition was detected for this age [F(2,44) = 31.171, *P* < 0.001, eta = 0.586], which is depicted in Fig. 3B” (left). LSD post-hoc tests indicated that adolescent mice buried more marbles in the tea tree-scented bedding, compared to the unscented bedding (*P* = 0.002), with no differences observed between vanilla-scented bedding and either the unscented (*P* = 0.235) or tea tree-scented conditions (*P* = 0.147). For the adults, a significant Sex × Bedding Condition interaction was observed [F(2,44) = 8.387, *P* < 0.001, eta = 0.276], which is depicted in Fig. 3B” (right). As illustrated, this interaction was driven by the male adult mice as tests for simple effects with LSD corrections did not detect a significant difference in the time spent burying by female mice in either the vanilla- (*P* = 0.052) or tea-tree-scented bedding (*P* = 0.213), compared to the unscented condition, while adult males spent more time burying in the tea tree-scented bedding versus both the unscented (*P* = 0.002) and vanilla-scented bedding (*P* = 0.009).

The significant 3-way ANOVA was then deconstructed along the Sex factor to explore age-related differences within each bedding environment within each sex. For females, the Age × Bedding Condition interaction was significant [F(2,44) = 14.136, *P* < 0.001, eta = 0.391]. As illustrated in Fig. 3B‴ (left), subsequent analysis for simple main effects with LSD post-hoc corrections revealed that adolescent females spent more time burying in the tea tree-scented bedding, compared to both the unscented (*P* < 0.001) and vanilla-scented conditions (*P* < 0.001), with no difference in burying time between unscented and vanilla conditions (*P* = 0.339). In contrast, the time spent burying did not differ across bedding conditions in adult females (*P*’s > 0.051). As illustrated in Fig. 3B‴ (right), the Age × Bedding Condition analysis of the data for males indicated a main Bedding effect only [F(2,44)-21.336, *P* < 0.001, eta = 0.492] that reflected a longer time burying by male mice in the tea tree-scented bedding, compared to both the vanilla-scented (*P* < 0.001) and unscented condition (*P* < 0.001). These data for the time spent burying indicate that while both scented bedding conditions can augment burying behavior in adult and adolescent mice, tea tree odor produces a much more robust effect, consistent with its known aversive property.

#### Number of marbles buried

A summary of group differences in the number of marbles buried in Experiment 2 is provided in Fig. 3C. An Age × Sex × Bedding Condition mixed-model ANOVA was conducted to examine for group differences in the number of marbles buried under the different scented conditions. The ANOVA results indicated no significant main effects of Age [F(1,44) = 0.001, *P* = 0.982, eta = 0.000] or Sex [F(1,44) = 2.48, *P* = 0.123, eta = 0.053], and no significant three-way interaction [F(2,88) = 0.595, *P* = 0.554, eta = 0.013]. A significant main effect was observed for Bedding Condition that is depicted in Fig. 3C’ [F(2,88) = 86.60, *P* < 0.001, eta = 0.663]. LSD tests for simple effects indicated that this Bedding Condition effect reflected more marbles buried under both the vanilla- (*P* = 0.027) and the tea tree-scented bedding (*P* < 0.001) versus the unscented condition, with mice also burying more marbles in the tea tree- than in the vanilla-scented bedding (*P* < 0.001). However, this effect varied as a function of age, as indicated by a significant Age × Bedding Condition interaction [F(2,88) = 3.725, *P* = 0.028, eta = 0.078]. Thus, this interaction was deconstructed along the Age factor to examine how the different bedding conditions influenced the number of marbles buried by adults and adolescent mice. As depicted in Fig. 3C”, analysis of simple main effects with LSD corrections revealed that both the adolescent and adult mice buried more marbles in the aversive-scented tea tree bedding when compared to both the neutral (*P* < 0.001 for both age groups) and unscented beddings (*P* < 0.001 for both age groups). Adolescent mice also exhibited increased burying behavior in the neutral vanilla bedding, compared to the unscented condition (Fig. 3C”; *P* = 0.049). In contrast, adult mice showed no significant difference in marble burying between the neutral and unscented beddings (Fig. 3C”; *P* = 0.231), indicative of an age-related difference in the response to the olfactory cues present in the bedding.

#### Distance traveled

A summary of group differences in the distance traveled during the 20-min marble-burying sessions in Experiment 2 is provided in Fig. 3D. We also employed an Age × Sex × Bedding Condition mixed-model ANOVA to investigate the locomotor activity of the mice, as measured by the distance traveled (in m) during the 20-min marble-burying testing session. Analysis of the main effects failed to indicate a significant effect of Sex [F(1,44) = 3.34, *P* = 0.074, eta = 0.071]. However, significant effects of Age (Adult > Adolescent; Fig. 3D’, left) [F(1,44) = 7.91, *P* = 0.007, eta = 0.152] and Bedding Condition (Fig. 3D’, right) [F(2,88) = 31.44, *P* < 0.001, eta = 0.417] were detected. LSD post-hoc tests for simple effects indicated that the Bedding Condition effect reflected a larger distance traveled in the unscented bedding versus both the vanilla- (*P* < 0.001) and tea tree-scented bedding (*P* < 0.001), in addition to a larger distance traveled in the vanilla- versus tea tree-scented conditions (*P* = 0.001). Additionally, an interaction between these factors was also detected [F(1.67, 73.25) = 17.616, *P* < 0.001, eta = 0.286] that is depicted in Fig. 3D”. Simple main effects analysis with LSD post-hoc corrections indicated that adolescent mice exhibited a marked reduction in locomotor activity when tested in the aversive tea-tree scented environment compared to the neutral vanilla and unscented conditions (Fig. 3D”: both *P*’s < 0.001). In contrast, adult mice exhibited lower locomotion when tested in both scented conditions relative to the unscented bedding control (Fig. 3D”; both *P*’s < 0.001), although they did locomote more in the aversive tea-tree condition versus the neutral vanilla bedding condition (*P* = 0.004). No significant three-way interaction of Age × Sex × Bedding Condition was detected for the distance traveled in Experiment 2 [F(1.67, 73.25) = 17.616, *P* < 0.001, eta = 0.286]. These data provide further evidence that locomotor activity is inversely related to marble-burying behavior, which likely reflects their behavioral incompatibility.

### Experiment 3: investigating aversive-conditioning following testing in the presence of an aversive odor

Considering the anxiogenic effect of exposure to tea tree oil observed in Experiment 2, we tested the hypothesis that initial exposure to tea tree odor might result in aversive-conditioning and increase subsequent marble-burying in the presence of clean, unscented, bedding. To this end, half of the mice underwent marble-burying procedures in the presence versus absence of the tea tree odor on day 1. The next day, all mice were tested under the standard, unscented condition to examine for carry-over effects as depicted in Fig. 1C. Scoring of behavior was conducted manually for Experiment 3 due to a computer malfunction, precluding replay of behavior to determine the latency to bury, the time spent burying and the collection of locomotor activity data. Thus, only the data for the number of marbles buried is presented.

#### Number of marbles buried

**Figure 4:**
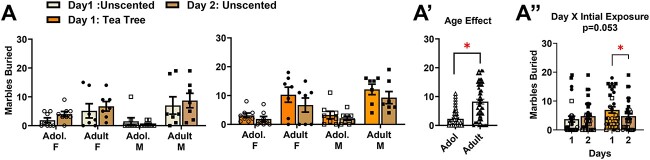
Examination of carry-over effects of a novel noxious chemosensory stimulus on the marble-burying behavior of adolescent and adult mice (Experiment 3). (**A**) Summary of the number of marbles buried by adolescent (Adol.) and adult male (M) and female (F) mice in the presence of unscented bedding (left) or tea tree-scented bedding (right) on day 1 of testing, and their response on day 2 of testing in the presence of unscented bedding only, the analyses of which yielded a strong trend towards a Day × Initial Exposure interaction [Adol. *n’s* = 8/sex; Adults *n’s* = 7/sex]. (**A**’) Depiction of the significant Age effect for the number of marbles buried. (**A**”) Depiction of the strong statistical trend for a Day × Initial Exposure interaction. The bar graphs represent the means ± SEMs of the number of individual mice indicated above. *P < 0.05, Initial Exposure effect (t-test)

A summary of the group differences in the number of marbles buried by the mice in Experiment 3 are presented in Fig. 4A. The data were analyzed using a Day (day 1 vs. day 2) × Age (adolescent vs. adult) × Sex (female vs. male) × Initial Exposure (unscented bedding vs tea-tree scented bedding) ANOVA. This ANOVA indicated a significant main effect of Age (Adult > Adolescent; Fig. 4A’) [F(1,42) = 25.06 *P* < 0.001, eta = 0.374], whereas the main effects of Day [F(1,52) = 0.98, *P* = 0.327, eta = 0.023, Sex [F(1,42) = 0.19, *P* = 0.665, eta = 0.005], and Initial Exposure [F(1,42) = 3.45, *P* = 0.070, *P* = 0.076] were not statistically significant.

Although the initial 4-way ANOVA failed to yield a significant interaction [F(1,42) = 0.057, *P* = 0.812, eta = 0.001], the data revealed a strong trend for a Day × Initial Exposure interaction [F(1,42) = 3.966, *P* = 0.053, eta = 0.086]. While we acknowledge that this interaction was not statistically significant, the large effect size prompted us to explore the Day × Initial Exposure interaction. For this, the data was collapsed across both ages and sexes prior to its deconstruction along the Initial Exposure factor to examine how the initial exposure to tea tree oil impacted marble-burying the next day. As there were only 2 levels on the Day factor, t-tests were employed for this follow-up analysis. A comparison of the number of marbles buried by mice exposed twice to unscented bedding detected no change in marbles buried [Fig. 4A”; t(24) = 0.819, *P* = 0.421]. In contrast, the number of marbles buried decreased when tea tree-exposed mice were allowed to bury marbles in unscented bedding (Fig. 4A”) [t(24) = 2.083, *P* = 0.048]. This later result suggests that the anxiogenic effect of tea tree oil on the number of marbles buried does not persist, arguing against the formation of a conditioned place-aversion to the general cage testing environment (to include sawdust bedding).

## DISCUSSION

Our laboratory has spent nearly a decade examining age by alcohol interactions in the manifestation of negative affect during alcohol withdrawal, with most studies employing male mice exclusively e.g. [15–17, 22–28]. In these studies, we reliably report: (1) increased indices of anxiety-like behavior in adult mice during early (24 h) alcohol withdrawal; (2) an incubation of anxiety-like behavior in adolescent mice during protracted withdrawal; and (3) higher basal anxiety-like behavior in water-drinking adolescent versus adult controls [22–28]. However, the gender gap in binge-drinking is closing, particularly among adolescents [55, 56] and the trajectory for developing an AUD occurs at a more rapid rate in alcohol-drinking women than men [2–5], with women [6–10] and adolescent girls [57, 58] exhibiting higher rates of comorbid psychiatric conditions, including affective disorders than age-matched males [6–10]. Thus, in recent years we have attempted to examine how biological sex moderates age-related differences in alcohol withdrawal-induced negative affect [15–17]. In contrast to our earlier studies, our more recent studies employing both sexes have failed largely at replicating our key observations [15–17]. Based on the results of a study by Kalaviers et al. [44], indicating that female odors can have an anxiolytic effect on adult males exposed to predator threat, we questioned whether socially relevant, sex-related, olfactory cues might be masking or interfering with our ability to detect age-related differences in both basal and alcohol withdrawal-induced anxiety-like behavior. The present study was designed to begin to address this question by assaying for the influence of sex-related odors on marble-burying behavior in alcohol-naive male and female, adult and adolescent, mice Below we discuss the present results in the context of the limited literature pertaining to the effect of innately motivating and novel benign olfactory cues on anxiety-like behavior in rodents.

Although female-soiled bedding did not influence the total number of marbles buried by the adult (PND 56–70) male mice in the present study, adult males exhibited a longer latency to begin, and a shorter time spent, burying marbles in the presence of female-related odors. These observations are interpreted as an anxiolytic effect of female pheromones in adult male mice. Such findings are in-line with the results of an earlier study of predator odor avoidance [44], in which sexually inexperienced adult male mice showed fewer fear responses and less predator odor avoidance when pre-exposed (either 1 min or 30 min) to female odors. In the present study, all mice (including the mice from which the soiled bedding was obtained) were sexually naïve, and none of the mice had ever been directly exposed to a conspecific of the opposite sex. Thus, the anxiolytic effect of female odors on the behavior of sexually naïve males extends across at least two very distinct paradigms of affective motivation and it will be important to determine if female-related chemicals, whether volatile or non-volatile, exert similar anxiolytic effects in other popular assays of negative affect in male rodents (e.g. elevated plus maze, light–dark shuttle box, forced swim and tail suspension tests). Gaining a clearer understanding of these effects would be of significant relevance to the experimental design and procedural timeline as well as the interpretation of findings from similar studies.

Admittedly, we did not monitor the estrous cycle of the females that generated the soiled bedding in this study. However, the female-soiled bedding was collected from multiple cages of group-housed females following a 6-day period and, thus, likely contained volatile and non-volatile chemicals emitted across all stages of the estrous cycle. Given our bedding collection procedure, it remains to be determined whether the apparent anxiolytic effect of the female-soiled bedding in adult males reflects a response to pheromones associated with female sexual receptivity that are innately motivating, e.g. [59, 60]. However, it should be noted that in the prior predator odor avoidance study by Kalaviers et al. [44], odors from sexually receptive and non-receptive females evoked equivalent anxiolytic effects in sexually naïve males. Thus, the anxiolysis observed in adult sexually naïve males in the presence of female odors may be independent of the females’ estrous phase, sexual receptivity and/or sexual experience. Regardless of the specific female-related pheromones driving the anxiolytic effect of female-soiled bedding in adult males, the reduction in marble-burying does not likely reflect a novelty-induced suppression of burying behavior. This conclusion is based on the observation that adult males exhibited a shorter, not longer, latency to marble-bury in the presence of both the novel neutral vanilla and novel noxious tea tree odors in the follow-up studies.

In Experiment 1, adult males buried the most marbles when exposed to bedding soiled by unfamiliar males. As the male-soiled bedding was obtained from group-housed males, it is very likely that it contained a blend of pheromones from socially dominant, intermediate, and subordinate mice. However, male mice are highly territorial, e.g. [61], and indicate their territorial boundaries using urinary scent markings, e.g. [62], with socially dominant males making urine marks in more locations and counter-marking more often than more subordinate males [63]. Thus, it can be presumed that of the four mice in each cage from which the male-soiled bedding was obtained, the majority of urine marks/pheromones present were those of the more dominant males. Based on the social dominance literature, e.g. [61–64], such a scenario should elicit a negative affective state related to a potential threat of a resident attack, particularly given the inescapable nature of the marble-burying apparatus. As we did not establish social hierarchy in our test subjects, it remains to be determined whether: (1) the greater propensity of males to bury marbles in male-soiled bedding reflects the relative amount of urine markings from dominant versus more subordinate males and (2) the social dominance of the male test subjects might dictate their response to the marbles in the presence of male- and/or female-soiled bedding, which could contribute to behavioral variability.

Although the anxiolysis exhibited by adult males in the presence of female-soiled bedding clearly does not require sexual experience to manifest, it does appear to depend upon their sexual maturity. This conclusion is based on the observation that young adolescent males (PND 27/28) exhibited higher, not lower, marble-burying in the presence of the female-soiled bedding. In fact, the magnitude of marble-burying by both adolescent males and females exposed to female- versus male-soiled bedding was equivalent. It was expected based on the social dominance literature, e.g. [64], that adolescent mice of both sexes might exhibit more signs of anxiety-like behavior in response to an inescapable environment containing the odors/pheromones of more dominant, mature adult males. However, we did not necessarily anticipate higher signs of negative affect in adolescent males or females presented with female-soiled bedding. While the literature concerning female dominance and aggression is rather limited, c.f., [65], female rats housed in same-sex colonies will determine dominant females that will attack any male intruder [66], and social dominance among female Syrian hamsters is predicted by body weight in the context of both female–female and female–male interactions [67]. Furthermore, adult female mice will spend more time attacking younger versus older intruders [68]. Thus, the increased marble-burying exhibited by both adolescent males and females in the presence of female-soiled bedding could be driven by the potential threat of being attacked by an older resident female mouse.

Alternatively, the high level of marble-burying of the adolescent males and females in response to both male- and female-soiled bedding may reflect their general responsiveness to novel odors. Although all mice exhibited a shorter latency to begin marble-burying in the presence of the neutral vanilla odor, only the adolescent mice buried more marbles in this context relative to the unscented, standard condition. Based on prior studies of the innate motivating properties of the two novel scents employed in this study [40, 50, 51], the number of marbles buried by adolescent mice in the presence of the vanilla odor was significantly less than that in the presence of the noxious tea tree odor, arguing that the tea tree odor is more anxiogenic than the vanilla odor in adolescent mice. Nevertheless, while age-related differences in marble-burying under the standard, unscented condition were not consistently detected across our three experiments, they did manifest in the presence of a male- and female-related odors, as well as a novel neutral odor. This suggests that the olfactory context is an important determinant of the ability to detect subject factor effects and interactions in this assay. Conversely, the fact that all mice exhibited similarly robust increases in marble-burying behavior in the presence of tea tree oil raises the possibility that the presence of novel noxious odorants (plant-based or otherwise) might mask the ability to detect subject factor differences and interactions in behavior. An important consideration for future research is whether our findings with the novel odorant extend to chemicals employed to sanitize the test cages and/or mask the scent of other test subjects [e.g. 70% (v/v) ethanol solutions or virucidal agents].

As observed in adult males, the presence of bedding soiled by same-sex conspecifics increased marble-burying in adult female mice as indicated by more time spent burying and more marbles buried, relative not only to the standard, unscented condition, but also to the male-soiled condition. Given the evidence for a social dominance hierarchy in group-housed female rats [66], it is possible that the high marble-burying exhibited by adult females in the presence of female-soiled bedding might be driven by the potential threat of being attacked by a more dominant conspecific. Alternatively, the adult female response might reflect the threat of resource competition emanating from the collection of pheromones from multiple unfamiliar female mice. Competition under conditions of limited resources (incl., food, water, nesting materials, sexual partners) is known to increase aggressive tendencies toward other females in rodents [66–68], but it is not known whether the threat of resource competition might induce a negative affective state in either female or male mice. As females are attracted to testosterone-dependent volatiles in male urine that indicate social status [69–71], it is perhaps not surprising that male-soiled bedding elicited a less robust effect on marble-burying by adult female mice than female-soiled bedding.

The facts that odors from same-sex adult conspecifics and odors from older animals increase marble-burying in adult and adolescent mice, respectively, in a manner akin to a noxious odorant speaks to the powerful influence exerted by chemosensory cues on anxiety-related behavior, particularly when studying same-sex cohorts of animals. That odors related to the opposite sex exert effects on the baseline behavior of adult mice that either are weaker (for females) or opposite (for males) to those effects produced by same-sex conspecifics also demonstrates that chemosensory stimuli shift baseline behavior in a sex- and age-dependent manner. Although the present study is not explicitly designed to delineate the nuanced impacts of social chemosensory stimuli on alcohol-induced changes in marble-burying behavior, it is relevant for interpreting the negative results from our prior studies of binge-drinking and negative affect [15, 16]. Specifically, our current results highlight the potential for social chemosensory cues, and possibly novel noxious odors from cleaning products, to minimize, completely obscure, or even facilitate the detection of subject factor differences, at least in the marble-burying assay.

## CONCLUSIONS

Behavior expressed in the marble-burying test is influenced by the presence of odors in the test environment. and the direction and magnitude of the influence of sex-related odors from both males and females, novel neutral odors (e.g. vanilla scent) and novel noxious odors (e.g. tea tree oil) varies as a function of the biological sex and/or the developmental age of the mouse.

## STUDY FUNDING

Funding for this project provided by NIH/NIAAA grant AA024044 to K.K.S and a National Science Foundation Pre-Doctoral Fellowship (award number 2139319) to C.L.J.C. .

## APC FUNDING

APC funding provided by NIH/NIAAA grant AA024044 to K.K.S.

## AUTHORS’ CONTRIBUTIONS

C. Leonardo Jimenez Chavez (Conceptualization [equal], Data curation [lead], Formal analysis [lead], Funding acquisition [supporting], Investigation [equal], Visualization [lead], Writing—original draft [equal], Writing—review & editing [equal]), and Karen Szumlinski (Conceptualization [equal], Data curation [equal], Formal analysis [supporting], Funding acquisition [lead], Investigation [supporting], Methodology [equal], Project administration [lead], Supervision [lead], Visualization [equal], Writing—original draft [equal], Writing—review & editing [equal])

## CONFLICT OF INTEREST STATEMENT

None declared.

## DATA AVAILABITY

Raw data will be made available upon request.

## ETHICAL STANDARDS

This work was conducted in an ethical manner with all experimental procedures in compliance with The Guide for the Care and Use of Laboratory Animals (National Research Council, 2014) and approved by the Institutional Animal Care and Use Committee of the University of California, Santa Barbara under protocol 862.2 and 862.3.

## Supplementary Material

Review_file_kvae009
