# Supplementary material for: Modulation of marble-burying behavior in adult versus adolescent C57BL/6J mice of both sexes by ethologically relevant chemosensory stimuli
Source: Oxf Open Neurosci. 2024 May 24;3:kvae009. doi: 10.1093/oons/kvae009 (PMC11195632; doi:10.1093/oons/kvae009)
Supplement: Review_file_kvae009 [file review_file_kvae009.zip › Review_file_kvae009.odt]

Round One Decision Letter, Reviews and Response:-

Manuscript ID OXFNSC-2023-015 entitled "Modulation of marble-burying behavior in adult versus adolescent C57BL/6J mice by ethologically relevant chemosensory stimuli" which you submitted to the Oxford Open Neuroscience, has been reviewed. The comments of the reviewer(s) are included at the bottom of this letter.

Please note that this journal operates with transparent peer review. This means that if your submission is accepted for publication, the full peer review history of your article will publish online alongside your article. This includes reviewer comments, editor decision letters, and your author responses.

The reviewer(s) have recommended publication, but also suggest some major revisions to your manuscript. Therefore, I invite you to respond to the reviewer(s)' comments and revise your manuscript within the next 60 days from today date.

To revise your manuscript, log into https://mc.manuscriptcentral.com/oxfnsc and enter your Author Centre, where you will find your manuscript title listed under "Manuscripts with Decisions."  Under "Actions," click on "Create a Revision."  Your manuscript number has been appended to denote a revision.

You may also click the below link to start the revision process (or continue the process if you have already started your revision) for your manuscript. If you use the below link you will not be required to login to ScholarOne Manuscripts.

*** PLEASE NOTE: This is a two-step process. After clicking on the link, you will be directed to a webpage to confirm. ***

https://mc.manuscriptcentral.com/oxfnsc?URL_MASK=72bc27388d8e4a78a783c2a1fc9718a6

You will be unable to make your revisions on the originally submitted version of the manuscript.  Instead, revise your manuscript using a word processing program and save it on your computer.  Please also highlight the changes to your manuscript within the document by using the track changes mode in MS Word or by using bold or colored text.

Once the revised manuscript is prepared, you can upload it and submit it through your Author Centre.

When submitting your revised manuscript, you will be able to respond to the comments made by the reviewer(s) in the space provided.  You can use this space to document any changes you make to the original manuscript.  In order to expedite the processing of the revised manuscript, please be as specific as possible in your response to the reviewer(s).

IMPORTANT:  Your original files are available to you when you upload your revised manuscript.  Please delete any redundant files before completing the submission.

Because we are trying to facilitate timely publication of manuscripts submitted to the Oxford Open Neuroscience, your revised manuscript should be uploaded within the next 60 days from today date.  If it is not possible for you to submit your revision in a reasonable amount of time, we may have to consider your paper as a new submission.

Once again, thank you for submitting your manuscript to the Oxford Open Neuroscience and I look forward to receiving your revision.


Reviewer: 1

Comments to the Author
The article provides a very rounded examination of the marble-burying behavior and the different variables that might affect it through 3 experiments considering sex, age and the type of stimuli used (bedding). Authors report differences in all three variables depending on the specific conditions of testing, with suggested nuance and closer examination when designing tasks that use the marble-burying test. Considering how common the test is, I believe the results reported are relevant for many researchers and deserve to be published. Behavior neuroscience without careful design of the behavioral task may lead to erroneous conclusions, so I deeply appreciate the authors' effort to not overlook the complexity of animal behavior.

The experiments seem well-designed. However, I found some issues related to how the results were reported that made it difficult to discern if the conclusions of each experiment are appropriate. Please see my main comments below:

1. The introduction requires refinement; the transition between the initial three paragraphs is abrupt, making it challenging to discern the connection among the discussed topics. While coherence emerges after perusing the experiments and results, dedicating effort to establishing seamless connections between these topics would enhance reader comprehension.
2. Mice were group-housed in 2-4. Given the existing literature on 'enrichment' concerning addictive and anxiety-related behaviors, one might consider the influence of housing the animals individually. A brief commentary on housing conditions as a potential influencing factor is warranted.
3. The Materials and Methods section could benefit from some figures/diagrams, such as: 1) a visual representation of the experimental setup, and 2) a table or diagram detailing the counterbalancing of conditions. Those figures could be referenced later during the results section, obviating the need to revisit the procedure section.
4. I am concerned regarding the methodology described in lines 24-26, where the entire cage is sprayed with essence (vanilla or tree oil). Given rodents' keen olfactory sensitivity, spraying only the bedding may have been sufficient. Additionally, clarity on box cleaning procedures, especially the removal of residual odors from polycarbonate cages, would help to eliminate those concerns.
5. Why did the authors decide on an LSD post-hoc comparison instead of a more traditional Tukey or Bonferroni correction?
6. The results section needs to be substantially revised. The current presentation lacks clarity on the specific statistical tests undertaken and their rationale. This makes it hard to estimate if the conclusions are appropriate. Furthermore, there are many inconsistencies in how the statistical tests are reported in terms of the amount of information or indicating which test was used. Please revise the results section. In the next comments, I provide more specific comments that are issues for all three experiments.
7. Regarding the 'deconstruction' of mixed-ANOVA in the methods section, caution is advised. The current approach seems inclined towards significant p-values, a practice that may benefit from reconsideration.
8. The results section starts by repeating the logic of each experiment. This seems redundant in most cases and is missing some of the rationale for the analyses. For instance, clear predictions.
9. While mixed ANOVAs are appropriate for the experiments, their reporting lacks precision. The authors should commence by presenting main effects for each variable before detailing interactions.
10. Related to the previous point, I would suggest indicating the levels of each variable to remind the reader how many Age and Bedding conditions there were (since those varied between each experiment).
11. Discrepancies in reporting p-values across the results section require rectification. Consistency with the specified significance level of 0.05 should be maintained.
12. Standardizing the amount of information pertaining to statistical analyses, as exemplified on page 5, line 13, is recommended for uniformity.
13. Perhaps because of the inconsistencies in the stats mentioned in the previous comment, it is not clear which statistical analysis was used for each of the ‘deconstructed’ analyses. For instance, on page 5, line 18 “p= 0.269,” it is impossible to know what the result means. Where is that p-value coming from?
a. From Experiment 1, Latency to bury: Please check that the conclusion in the last two lines is correct. Authors found that females buried quicker when scented vs. non-scented, whereas males took longer when female bedding was used. If I understood from their introduction, burying quicker indicates more anxiety (which is what females did), but authors conclude the opposite. Please revise. I do believe that when authors rewrite their stats reporting it will also help with this.
b. In Exp 1- Total time spent burying, there is no conclusion about what the results mean.
c. It is not clear to me why the authors decided to ‘re-run’ the ANOVA removing some variables. Instead, post-hoc should be run for each main effect and interaction found.
d. In Exp 1- Number of marbles buried: This section is confusing. It is not clear how and why some analyses were done. I do not understand why the authors “deconstructed” the analysis across the Age factor when there was a 3-way interaction. In this same section, revise the consistency of p-value reporting.
e. In Exp 1- Number of marbles buried on page 6: Once again, it is unclear why some ANOVAs were ‘reanalyzed’ (is reanalysis the same as deconstructed?). Post-hocs should be used or provide logic on why some variables are pooled. Also, please check if your result justifies reanalyzing data, or if the factor should be added as a covariate.
f. Distance traveled: This is pretty interesting but was not mentioned during the methods section that it was going to be analyzed. Also, it seems like a missed opportunity to only report for Experiment 1 but not Experiments 2 or 3.
g. For Experiment 2, please provide evidence for the rationale not to counterbalance the tea tree odor. Where is that concern coming from?
h. Related to the previous one, if the fear was place-aversion, wouldn’t it make sense to not test all animals in the same context/box? I think it is not clear where the animals were tested. This concern also seems to contradict the decision to spray the box and not only the bedding with the essence.
i. In Experiment 3, an explanation is needed for anticipating carry-over effects. The rationale behind this test's inclusion was not obvious.
j. In Experiment 3, incorporating latency or distance measures, akin to Experiments 1 or 2, would enhance comparability and completeness.
14. In the discussion section, I think it needs to become more evident what contradictions the authors have observed in their own research to motivate this study.
15. The phrasing of the opening paragraph needs to be revised. The last two lines are unclear or need more context.
16. Page 8, line 4-5. It is true that the NIH requests to include sex as a biological variable. However, it is more important to express why this is relevant and overdue for the scientific community. I believe that line should be removed and replaced with the relevance of studying both sexes in research. Furthermore, this research might seem new to the neuroscience community; however, animal behavior researchers from a more ecological or even Pavlovian/Operant conditioning background have highlighted the influence of pheromones in animal behavior before. Perhaps some references could be added or the phrasing should be revised.

Minor comments:
- Page 2, Line 46-47: Delete the parenthesis with “put in the manufacturer of the bedding; I can take a photo today when I go to the lab”.  
- Page 5, line 10: What do you mean by reactivity?
- Page 5, line 13: Why is Condition capitalized? Do you mean Condition is a variable? If so which one
- Page 5, line 38: Revise if ‘assayed’ is the word intended to use there.
- Page 5, line 38-39: the writing is odd and difficult to understand
- Page 6, line line 8: “See Above”, please explain where in text.
- Page 6, line 37: When you state “to facilitate the interpretation of the results in Experiment 1” please explain how it would facilitate the understanding of the results. I think this could be more obvious if predictions were provided.

Reviewer: 2

Comments to the Author
The authors previously demonstrated a reliable age-dependent (adolescent versus adult) difference in basal and alcohol withdrawal-induced negative affect measured with the marble-burying test in male mice. However, since they included female mice in their cohort of animals, they are not able to replicate their own data or obtained opposite effects. Therefore, they decided to conduct this study to understand how sexual pheromones can modulate marble-burying behavior in adolescent and adult drug-naïve male and female mice. Because of the necessity and the requirement to include females to vertebrate animal studies, this project addresses an important question and highlights the importance to understand and adapt the behavioral models to both sexes. While the question is timely and will bring important knowledge to the field, the way the results are represented makes it difficult to understand the data and break the flow of the manuscript.

Major comments
1) The authors designed their experiments with many parameters, the age, sex and bedding which brings a lot of comparisons and information. Therefore, the representation and analysis of the results should be revised to clarify the message. For example, Figure 1C’ left shows the same results as Figure 1C with a focus on sex and adults whereas Figure 1C’ right collapses data of all adolescents independently of the sex which make difficult to follow and understand the results. The authors should clarify the question/hypothesis they want to answer/test and reorganize the results accordingly to make their message clearer to the readers. The same applies to Experiments 2 and 3.

2) The conclusion of the results lines 21 to 23 should be reviewed. The authors interpreted the results from Figure 1A’ which collapses both ages, therefore, it cannot be concluded that female ordors exerts an anxiolytic effect in both adult and adolescent males but they exert and anxiolytic effects in males independently of the age.

3) In the first experiment, the authors highlighted a decrease of the locomotion in all mice for the female- and male-soiled bedding. It could suggest that the presence of pheromones induced an adaptation of the behaviors from active to passive strategy. Did other types of behaviors such as freezing, immobility change during the test between the groups and conditions?

4) While the authors report 3 behaviors (the burying latency, the burying time and the marbles buried) in experiment 1, they only report partially these behaviors in experiment 2 and 3. An analysis of these 3 behaviors for the 3 experiments will provide more robust understanding of the role of sexual pheromones on the marble burying behavior in both sexes. If these 3 behaviors reveal inconsistent results between each other it should be discussed.

5) With regard to the interpretation, in experiment 2 the authors report a decrease of the burying latency in the presence of aversive or neutral bedding compared to the unscented bedding independently of the sex and the age of the mice. However, the marbles buried analysis revealed an age-dependent effect of the bedding. How the authors reconcile these different results?

Minor comments:
1) The title should be reviewed to state that the study compare/study both male and female mice.
2) The authors should read carefully the manuscript to remove some typos/comments through the manuscript. To site one: lines 46-47 remove “(put in the manufacturer of the bedding; I can take a photo today when I go into the lab)”

Associate Editor
Comments to the Author:
Methods
- The authors clearly state their exclusion criterion in the methods but the exact number of exclusions per experiment should be stated. It also appears that mice may have been excluded from one measure (e.g., latency to bury in Expt 1) but not other measures (e.g., total time spent burying). Please provide more details on these exclusions.
- Line 33/34: it is stated n=8 adolescent mice/sex but in Figure 3 it is stated that there are 10 adolescent mice per sex.

Figure captions
- I believe there is an error in the Figure 1 caption: for A', females mice exhibited a shorter (not longer) latency to bury in the presence of soiled beddings compared to unscented bedding.
- Figure 3 caption: please keep the variable names consistent. Here the factor "bedding" was used while in the results section it was termed "initial exposure". Also, the Day x Bedding interaction only approached significance and this should be acknowledged in this caption.

Response to reviewers:-
We would like to begin by thanking both reviewers very much for their efforts in providing feedback on our report. In this revision, we have attempted to the best of our ability to address or respond to the comments/concerns of both reviewers and, in our opinion, the report is much improved thanks to their input. Please find below a detailed response to the reviewers’ comments and all changes to the text are highlighted in yellow to facilitate detection of changes.

Reviewer 1 comments:

1. The introduction requires refinement; the transition between the initial three paragraphs is abrupt, making it challenging to discern the connection among the discussed topics. While coherence emerges after perusing the experiments and results, dedicating effort to establishing seamless connections between these topics would enhance reader comprehension.

Reply: We have reworked the Introduction as suggested by the reviewer. We think that the revised Introduction provides a clearer rationale for the studies, which should address some of the additional reviewer concerns below.

2. Mice were group-housed in 2-4. Given the existing literature on 'enrichment' concerning addictive and anxiety-related behaviors, one might consider the influence of housing the animals individually. A brief commentary on housing conditions as a potential influencing factor is warranted.

Reply: We are unclear why the reviewer would like us to discuss group vs. isolated housing as this is not relevant to the current experiment or any of our prior studies of alcohol withdrawal. Given that we did not systematically manipulate housing conditions in our studies, we opted to refrain from discussing or commenting on housing condition in the revised report.

3. The Materials and Methods section could benefit from some figures/diagrams, such as: 1) a visual representation of the experimental setup, and 2) a table or diagram detailing the counterbalancing of conditions. Those figures could be referenced later during the results section, obviating the need to revisit the procedure section.

Reply: A depiction of the procedural time-lines for the different experiments is now provided as new Figure 1.

4. I am concerned regarding the methodology described in lines 24-26, where the entire cage is sprayed with essence (vanilla or tree oil). Given rodents' keen olfactory sensitivity, spraying only the bedding may have been sufficient. Additionally, clarity on box cleaning procedures, especially the removal of residual odors from polycarbonate cages, would help to eliminate those concerns.

Reply: We have described more carefully how the odorants were applied to the cages. The odorants were not sprayed all over the cage. The odorants were applied to a Kimwipe and then a 5-cm stripe was wiped around the cage wall as we have conducted previously in studies of predator odor exposure (Quadir et al., 2016, 2019). This procedure ensures uniform scent distribution and reduces concentrated localized odor sources that might be avoided or preferred by the mice. The cage cleaning procedures were provided in the original manuscript but have also been elaborated upon to provide more detail on our best attempt to remove all residual odors (see page 3). As described, the cage cleaning procedures are standard practice for neutralizing pathogens and odors.

5. Why did the authors decide on an LSD post-hoc comparison instead of a more traditional Tukey or Bonferroni correction?

Reply: We have now provided a rationale for our selection of the LSD post-hoc methods for detecting group differences (see page 4). Our study was designed with an exploratory framework aimed to discover potential effects that may modulate behavior. The LSD method, while less conservative than the more traditional corrections, offers greater statistical power, which aligns with the exploratory nature of our study.

6. The results section needs to be substantially revised. The current presentation lacks clarity on the specific statistical tests undertaken and their rationale. This makes it hard to estimate if the conclusions are appropriate. Furthermore, there are many inconsistencies in how the statistical tests are reported in terms of the amount of information or indicating which test was used. Please revise the results section. In the next comments, I provide more specific comments that are issues for all three experiments.

Reply: We are not entirely clear about why this reviewer thinks that the presentation of our results section lacks clarity in terms of the specific statistical tests employed. Throughout the results section, we clearly state that the data were analyzed using an Age by Sex By Bedding ANOVA (see for example, Latency to Bury on page 5) and guided the reader through the deconstruction of significant interactions.

7. Regarding the 'deconstruction' of mixed-ANOVA in the methods section, caution is advised. The current approach seems inclined towards significant p-values, a practice that may benefit from reconsideration.

Reply: We prioritize significant p-values because it is a common practice, but we recognize that there are risks associated with an overemphasis on p-values. So, to address this problem, we implemented several safeguards. (1) We maintained a standard alpha level (alpha = 0.05) and reported effect sizes (partial eta squared) to provide a more comprehensive understanding of the data. (2) We also added post hoc analyses to our ANOVAs. Please note that were there were p-values <.05 were typically associated with moderate to higher effect sizes. Unfortunately, the SPSS software (version 27 for Mac nor PC) does not allow for estimating effect sizes beyond overall ANOVA.

8. The results section starts by repeating the logic of each experiment. This seems redundant in most cases and is missing some of the rationale for the analyses. For instance, clear predictions.

Reply: Given that our 3 experiments employed different bedding conditions, we deemed it important to remind the reader of the specific manipulations at the outset of each results subsection to help guide interpretation of the findings. Indeed, in comment #10 below, this reviewer requests that we indicate the levels of bedding employed in each experiment as the bedding conditions differed. The entire point of the introductory sections to each experimental result was to remind the reader of the specific bedding conditions tested. We have carefully reviewed each introductory section to ensure that the experimental designs were properly described and we have provided specific predictions for these subsections as suggested by this reviewer.

9. While mixed ANOVAs are appropriate for the experiments, their reporting lacks precision. The authors should commence by presenting main effects for each variable before detailing interactions.

Reply: Originally, we did not include the details of significant main effects when significant interactions were detected as the interpretation of main effects is directed by/depends upon significant interacting factors and we wished to keep the results section as straight-forward as possible. However, for completeness, we have now inserted the results for the main effects throughout the report.

10. Related to the previous point, I would suggest indicating the levels of each variable to remind the reader how many Age and Bedding conditions there were (since those varied between each experiment).

Reply: We have now inserted the different levels of bedding in the first result as suggested. See also response to #8.

11. Discrepancies in reporting p-values across the results section require rectification. Consistency with the specified significance level of 0.05 should be maintained.

Reply: We would like to clarify that to the best of our knowledge, we did not refer to any findings with p-values above the specified alpha level of 0.05 as statistically significant. The inclusion of these p-values, even when above the conventional threshold for significance was intended to offer a comprehensive view of the data especially where data trends may be of interest, allowing for comparison between the significant and non-significant effects. When post-hoc tests indicated a difference between the unscented bedding condition and the other two conditions, we employed a summary p value that described the results of both comparisons to reduce the wordiness of the results (e.g., p’s < 0.01). In case the reviewer was referring to these instances, we have now included the specific p values for each comparison. There is only one instance when the results of our ANOVA indicated a strong trend for statistical significance and that occurred in Experiment 3 when the p value was 0.053. As justified as an exploratory analysis, we deconstructed this interaction and this is appropriate given the exploratory nature of this experiment.

12. Standardizing the amount of information pertaining to statistical analyses, as exemplified on page 5, line 13, is recommended for uniformity.

13. Perhaps because of the inconsistencies in the stats mentioned in the previous comment, it is not clear which statistical analysis was used for each of the ‘deconstructed’ analyses. For instance, on page 5, line 18 “p= 0.269,” it is impossible to know what the result means. Where is that p-value coming from?

Reply: We have carefully reviewed the presentation of results for all three experiments and worked hard to ensure consistency in reporting the results and to clarify when post-hoc test results are presented. Upon this review, we realized that we omitted the results of the one-way ANOVAs conducted upon the deconstruction of significant interactions and for that, we humbly apologize. We have now inserted those results and have clearly indicated which statistical approaches were employed to engender each statistical result/p value.

13a. From Experiment 1, Latency to bury: Please check that the conclusion in the last two lines is correct. Authors found that females buried quicker when scented vs. non-scented, whereas males took longer when female bedding was used. If I understood from their introduction, burying quicker indicates more anxiety (which is what females did), but authors conclude the opposite. Please revise. I do believe that when authors rewrite their stats reporting it will also help with this.

Reply: The concluding sentences from this subsection are: “Thus, irrespective of age, these data for the latency to first marble bury indicate that female odors exert an anxiolytic effect in male mice. While social chemosensory stimuli from both sexes are anxiogenic in females, as evidenced by their reduced latency to bury.” Thus, we are concluding that males are less anxious in the presence of female odors, while odors from both males and females increase anxiety-like behavior in females. We are not clear why the reviewer says we are concluding otherwise.

b. In Exp 1- Total time spent burying, there is no conclusion about what the results mean.

Reply: We have added a concluding sentence for time spent burying.

c. It is not clear to me why the authors decided to ‘re-run’ the ANOVA removing some variables. Instead, post-hoc should be run for each main effect and interaction found.
d. In Exp 1- Number of marbles buried: This section is confusing. It is not clear how and why some analyses were done. I do not understand why the authors “deconstructed” the analysis across the Age factor when there was a 3-way interaction. In this same section, revise the consistency of p-value reporting.
e. In Exp 1- Number of marbles buried on page 6: Once again, it is unclear why some ANOVAs were ‘reanalyzed’ (is reanalysis the same as deconstructed?). Post-hocs should be used or provide logic on why some variables are pooled. Also, please check if your result justifies reanalyzing data, or if the factor should be added as a covariate.

Reply: As explained in our Statistical Analyses section, our statistical method involves first running a 3-way mixed-model ANOVA between Age, Sex and Bedding Condition to identify any significant interaction or main effects. When a 3-way interaction is detected, the data are split along 2 of the factors (one at a time, in this study, Age and then Sex), followed by a 2-way ANOVA to examine for next-level interaction. This is the standard procedure to determine the source(s) of a 3-way interaction. Any ensuing significant 2-way ANOVAs or the initial detection of a 2-way ANOVA (upon the initial 3-way ANOVA) are followed up by splitting the data along a relevant factor, conducting tests for simple effects that if significant, are followed by determining specific group differences using LSD post-hoc tests. In cases where no significant interactions are found upon the initial 3-way ANOVA, we proceed to examine the main effects using LSD post-hoc tests on the data collapsed across the relevant groups.

f. Distance traveled: This is pretty interesting but was not mentioned during the methods section that it was going to be analyzed. Also, it seems like a missed opportunity to only report for Experiment 1 but not Experiments 2 or 3.

Reply: Distance traveled was mentioned in the original version of the report under the general description of marble-burying procedures. As now explained in the Methods (see page 4), a computer malfunction precluded determination of the latency to bury or the distance traveled in Experiment 3. However, we have now included the distance data for experiment 2 and these results can be found on page 8 and the data are included in new Figure 3C,C’. Unfortunately, the short turn-around time for the revision precluded our completion of an analysis of the time spent burying for Experiment 2.

g. For Experiment 2, please provide evidence for the rationale not to counterbalance the tea tree odor. Where is that concern coming from?
h. Related to the previous one, if the fear was place-aversion, wouldn’t it make sense to not test all animals in the same context/box? I think it is not clear where the animals were tested. This concern also seems to contradict the decision to spray the box and not only the bedding with the essence.
i. In Experiment 3, an explanation is needed for anticipating carry-over effects. The rationale behind this test's inclusion was not obvious.

Reply: Tree tea oil is aversive for mice but it’s ability to induce a conditioned place-aversion or a generalization of place-aversion was unknown at the time of study. As stated on page 4 (and eluded to in the reviewer’s comment “h”), we were concerned that introducing this aversive smell early in the experimental sequence could lead to conditioned aversion to the general test environment, which could manifest during subsequent testing as increased marble burying. Indeed, a single exposure to an aversive stimulus in a particular context is sufficient to induce a lasting aversion (Wiedenmayer, 2004), although few studies have studied this in the context of aversive smells, we wanted to avoid this problem. Given our demonstration of an aversive reaction to the tea tree odor at the end of Experiment 2, we wanted to determine once and for all whether such aversive conditioning actually occurs under our procedures. As discussed on page 4, not only do the results of Experiment 3 address a gap in the literature regarding the lasting impact of aversive olfactory experiences on anxiety-related behavioral test measures, they help in refining experimental designs by highlighting the importance of considering potential carryover effects in behavioral paradigms that employ repeated measures. See our response to why we chose to swipe the odor on the walls of the box in our response to #4. Arguably, the presence of tea tree odor in the bedding could have also induced a conditioned aversion to bedding, which would require a comparable follow-up investigation.

j. In Experiment 3, incorporating latency or distance measures, akin to Experiments 1 or 2, would enhance comparability and completeness.
Reply: See response to 14f above.

14. In the discussion section, I think it needs to become more evident what contradictions the authors have observed in their own research to motivate this study.
15. The phrasing of the opening paragraph needs to be revised. The last two lines are unclear or need more context.
Reply: We have completely reworked the introductory paragraph to the Discussion based on the reviewer’s suggestions.

16. Page 8, line 4-5. It is true that the NIH requests to include sex as a biological variable. However, it is more important to express why this is relevant and overdue for the scientific community. I believe that line should be removed and replaced with the relevance of studying both sexes in research. Furthermore, this research might seem new to the neuroscience community; however, animal behavior researchers from a more ecological or even Pavlovian/Operant conditioning background have highlighted the influence of pheromones in animal behavior before. Perhaps some references could be added or the phrasing should be revised.
Reply: We have removed the sentence regarding NIH. With the reworking of the Introduction and the introductory paragraph to the Discussion, we have provided a solid rationale for why our laboratory (and other laboratories) should include female subject.

Minor comments:
- Page 2, Line 46-47: Delete the parenthesis with “put in the manufacturer of the bedding; I can take a photo today when I go to the lab”.
Reply: Thank you so much for noticing this mistake! We have now included the details of the bedding manufacturer.

- Page 5, line 10: What do you mean by reactivity?
Reply: We have replaced the term “reactivity” throughout.

- Page 5, line 13: Why is Condition capitalized? Do you mean Condition is a variable? If so which one
Reply: “Condition” should have been “Bedding” and we have corrected.

- Page 5, line 38: Revise if ‘assayed’ is the word intended to use there.
Reply: Revised as suggested.

- Page 5, line 38-39: the writing is odd and difficult to understand
Reply: We divided the sentence in two to increase clarity.

- Page 6, line line 8: “See Above”, please explain where in text.
Reply: This was in reference to the aforementioned 3-way interaction. We have made this clearer in the manuscript.

- Page 6, line 37: When you state “to facilitate the interpretation of the results in Experiment 1” please explain how it would facilitate the understanding of the results. I think this could be more obvious if predictions were provided.
Reply: The notion that the results of Experiment 2 might inform the interpretation of the results of Experiment 1 was actually first mentioned in the Methods section on page 4. Thus, we have inserted an explanation there. We also provided predictions at the outsets of each results section (see response to #8).


Reviewer 2 comments:

Major comments
1) The authors designed their experiments with many parameters, the age, sex and bedding which brings a lot of comparisons and information. Therefore, the representation and analysis of the results should be revised to clarify the message. For example, Figure 1C’ left shows the same results as Figure 1C with a focus on sex and adults whereas Figure 1C’ right collapses data of all adolescents independently of the sex which make difficult to follow and understand the results. The authors should clarify the question/hypothesis they want to answer/test and reorganize the results accordingly to make their message clearer to the readers. The same applies to Experiments 2 and 3.

Reply: We have now included a logic for the presentation of our results on pages 4-5. For transparency, we show all of the data from our different groups in the left column of each figure. Then any follow-up main effects or interactions that cannot be readily derived from an examination of the data in the left columns are graphically depicted to the right. We have removed the graph for the adults from Figure 1C’ left because it is completely redundant with the data in Figure 1C and have now indicated specific differences between bedding conditions for the adult mice in Figure 1C proper.

2) The conclusion of the results lines 21 to 23 should be reviewed. The authors interpreted the results from Figure 1A’ which collapses both ages, therefore, it cannot be concluded that female ordors exerts an anxiolytic effect in both adult and adolescent males but they exert and anxiolytic effects in males independently of the age.

Reply: We agree and have corrected the conclusion and reviewed the other conclusions for similar mistakes.

3) In the first experiment, the authors highlighted a decrease of the locomotion in all mice for the female- and male-soiled bedding. It could suggest that the presence of pheromones induced an adaptation of the behaviors from active to passive strategy. Did other types of behaviors such as freezing, immobility change during the test between the groups and conditions?

Reply: Upon review of the video recordings there were no overt periods of immobility detected by our ANY-Maze software to suggest freezing behavior per se (sensitivity set to 5 sec of no displacement of the animal’s center of gravity) and a switch from active to passive coping. This being said, we also observed lower locomotor activity in the mice when exposed to the tea tree-scented bedding (new data now provided). Again, no overt freezing behavior was observed. Thus, as of now, we think that the apparent inverse relation between locomotion and burying may simply reflect the fact that burying behavior is physically incompatible with horizontal locomotion.

4) While the authors report 3 behaviors (the burying latency, the burying time and the marbles buried) in experiment 1, they only report partially these behaviors in experiment 2 and 3. An analysis of these 3 behaviors for the 3 experiments will provide more robust understanding of the role of sexual pheromones on the marble burying behavior in both sexes. If these 3 behaviors reveal inconsistent results between each other it should be discussed.

Reply: Indeed, a uniform assessment across all 3 experiments would offer a more holistic understanding. Regrettably, due to an unexpected malfunction of our video tracking system, we were unable to video record the sessions from Experiment 3 and consequently, this impedes us from providing further behavioral data for this experiment. This is now explained in the Methods and Results sections. We were able to playback the video recording from Experiment 2 to obtain distance data, which is now provided in new Figure 3C and C’. However, due to the limited amount of time available to revise this report, we were not able to complete our analyses of the time spent burying for Experiment 2.

5) With regard to the interpretation, in experiment 2 the authors report a decrease of the burying latency in the presence of aversive or neutral bedding compared to the unscented bedding independently of the sex and the age of the mice. However, the marbles buried analysis revealed an age-dependent effect of the bedding. How the authors reconcile these different results?

Reply: Over the past decade, we have often noticed distinctions in group differences for latency measures in our assays of negative affects (including, but not limited to, marble-burying) versus total measures over the course of the test (see Refs 22-28 for our data for alcohol withdrawal). One thing to note is that the latency measure reflects the initial response to the test environment (in this case, the marbles and test cage). This responsiveness can (or may not) change as the animal familiarizes itself with the test environment and begins to react to or respond within that test environment. While an “anxious” animal would be predicted to have a shorter latency to bury, in addition to a longer bury time and more marbles buried, we have learned over the years that, more often than not, latency effects are different from effects on time burying and number buried, presumably because these variables are independent. Notably, the vast majority of marble-burying studies in the literature only examine the number of marbles buried. Our attempts to provide a more thorough assessment of behavior sometimes bring with them dissociations that are difficult to reconcile at times.

Minor comments:
1) The title should be reviewed to state that the study compare/study both male and female mice.
Reply: We have revised the title to include sex.

2) The authors should read carefully the manuscript to remove some typos/comments through the manuscript. To site one: lines 46-47 remove “(put in the manufacturer of the bedding; I can take a photo today when I go into the lab)”
Reply: We apologize for our sloppiness and have carefully reviewed the manuscript for such omissions and typos.

Associate Editor
Comments to the Author:
Methods
- The authors clearly state their exclusion criterion in the methods but the exact number of exclusions per experiment should be stated. It also appears that mice may have been excluded from one measure (e.g., latency to bury in Expt 1) but not other measures (e.g., total time spent burying). Please provide more details on these exclusions.

Reply: Yes, the Editor is correct in that mice excluded from one variable may not have been excluded from a different variable depending on the results of the outlier analyses of the different variables. We have now detailed the number of animals removed and for which variable on page 5.

- Line 33/34: it is stated n=8 adolescent mice
e/sex but in Figure 3 it is stated that there are 10 adolescent mice per sex.

Reply: Thank you for catching this copy and paste error. We have corrected the sample sizes in the figure legend for new Figure 4.

Figure captions
- I believe there is an error in the Figure 1 caption: for A', females mice exhibited a shorter (not longer) latency to bury in the presence of soiled beddings compared to unscented bedding.
- Figure 3 caption: please keep the variable names consistent. Here the factor "bedding" was used while in the results section it was termed "initial exposure". Also, the Day x Bedding interaction only approached significance and this should be acknowledged in this caption.

Reply: Thank you for catching these mistakes.
Round Two Decision Letter, Reviews and Response:-
Manuscript ID OXFNSC-2023-015.R1 entitled "Modulation of marble-burying behavior in adult versus adolescent C57BL/6J mice of both sexes by ethologically relevant chemosensory stimuli" which you submitted to the Oxford Open Neuroscience, has been reviewed. The comments of the reviewer(s) are included at the bottom of this letter.

As you'll note, reviewer 1 is satisfied with the revisions, but reviewer 2 still suggests some major revisions to your manuscript. Therefore, I invite you to respond to the reviewer's comments and revise your manuscript within the next 60 days from today's date. The Associate Editor and I have also evaluated the revision and we disagree that an additional experiment is needed, but we do agree with the request for additional data analyses. Essential revisions should be focused on Experiment 3: time spent burying data and analysis should be included (even if it requires additional time for the revision) and more caution should be applied in the results and discussion section when interpreting the Day x Initial exposition interaction in Experiment 3 as it only shows a trend toward significance (p = 0.053). Here is some additional guidance in responding to reviewer 2's remaining concerns:

#1: Authors have clearly stated their reason for using the LSD post-hoc analysis. We believe this is fine.
#2: The deconstruction of the 3- or 4-way ANOVA is also fine, although authors should clarify this further in the text. The authors should also ensure that the figures are presented in a manner that best reflects the statistical analyses.
#3: We agree with reviewer 2 that the additional analyses should be included.
#4: We disagree with reviewer 2 that this experiment needs to be conducted again. 
#5: We agree in recommending more caution in the interpretation given that it relies on a statistical trend. This should also be acknowledged in the Discussion.

Please note that this journal operates with transparent peer review. This means that if your submission is accepted for publication, the full peer review history of your article will publish online alongside your article. This includes reviewer comments, editor decision letters, and your author responses.

To revise your manuscript, log into https://mc.manuscriptcentral.com/oxfnsc and enter your Author Centre, where you will find your manuscript title listed under "Manuscripts with Decisions."  Under "Actions," click on "Create a Revision."  Your manuscript number has been appended to denote a revision.

You may also click the below link to start the revision process (or continue the process if you have already started your revision) for your manuscript. If you use the below link you will not be required to login to ScholarOne Manuscripts.

*** PLEASE NOTE: This is a two-step process. After clicking on the link, you will be directed to a webpage to confirm. ***

https://mc.manuscriptcentral.com/oxfnsc?URL_MASK=d04636a20785499797baf44bb24ffdbf

You will be unable to make your revisions on the originally submitted version of the manuscript.  Instead, revise your manuscript using a word processing program and save it on your computer.  Please also highlight the changes to your manuscript within the document by using the track changes mode in MS Word or by using bold or colored text.

Once the revised manuscript is prepared, you can upload it and submit it through your Author Centre.

When submitting your revised manuscript, you will be able to respond to the comments made by the reviewer(s) in the space provided.  You can use this space to document any changes you make to the original manuscript.  In order to expedite the processing of the revised manuscript, please be as specific as possible in your response to the reviewer(s).

IMPORTANT:  Your original files are available to you when you upload your revised manuscript.  Please delete any redundant files before completing the submission.

Because we are trying to facilitate timely publication of manuscripts submitted to the Oxford Open Neuroscience, your revised manuscript should be uploaded within the next 60 days from todays date.  If it is not possible for you to submit your revision in a reasonable amount of time, we may have to consider your paper as a new submission.

Once again, thank you for submitting your manuscript to the Oxford Open Neuroscience and I look forward to receiving your revision.


Reviewer: 1

Comments to the Author
I appreciate the efforts of the authors to take into consideration most of the comments. I think the article has improved significantly. It is very easy to follow the logic of each experiment, its results, and their conclusions. I don’t have additional comments.


Reviewer: 2

Comments to the Author
I want to thank the authors for addressing the comments raised after the first submission. Nevertheless, some important aspects are still pending.

1) In the statistical section the authors justified the use of LSD post-hoc analysis instead of Tukey’s test as it offers greater statistical power to detect group differences. The justification should rely on a more scientific and/or statistical arguments.
2) As mentioned for the first submission the deconstruction of the 3/4 way-ANOVA remains unclear and unusual. Although the authors added in the statistical section the logic of the presentation, the figures/graphs should be revised.
3) The review from the first submission suggested to provide all the behavioral parameters analyzed during the marble-burying test for the 3 experiments to provide a more robust understanding of the role of sexual pheromones on affective behavior. For the experiment 2, the authors added the results of the distance travelled but report a lack of time to compute the time spent burying. We still believe that these results are necessary to the study and therefore should be included.
4) Moreover, for the experiment 3, the authors report a technical issue during the tests which did not allow to videotape the tests preventing to provide the latency to bury, the burying time and the locomotion. Technical issues are unfortunate, a replication of this experiment to collect all the data needed of the project would be welcome.
5) The statistical analysis of the experiment 3 needs to be reviewed. Indeed, the 4 way ANOVA did not yield significant interaction. However, the authors report a strong trend for the Day x Initial exposition interaction and then follow up with a t-test analysis. Statistical analysis on trend effect are difficult to interpret and should be taken with caution. Moreover, it supports my comment above suggesting that this experiment should be remade to get a complete picture of the behaviors observed under this experimental conditions.

Minor comments :
1) The authors should read carefully the manuscript to remove some typos/comments through the manuscript. Line 7 page 9 the end of the sentence seems to be missing. Same comment line 44 page 9.
2) To clarify the experimental design in Figure 1, the authors could add the symbol male or female on the back of the mice as the sex do not appear in the figure.

Response to Reviewers
We would like to begin by thanking both reviewers and the Editors very much for their efforts in providing feedback on our report. In this revision, we have attempted to the best of our ability to address the remaining concerns of Reviewer 2, using the guidance provided by the Editors. Please find below a detailed response to the reviewers’ comments and all changes to the text are highlighted in blue to facilitate detection of new revisions. Former revisions are still highlighted in yellow.

Reviewer 2 comments:

1) In the statistical section the authors justified the use of LSD post-hoc analysis instead of Tukey’s test as it offers greater statistical power to detect group differences. The justification should rely on a more scientific and/or statistical arguments.

Editorial guidance: Authors have clearly stated their reason for using the LSD post-hoc analysis. We believe this is fine.

Reply: Given the guidance put forth by the Editors, we have opted not to revise our reasoning any further.

2) As mentioned for the first submission the deconstruction of the 3/4 way-ANOVA remains unclear and unusual. Although the authors added in the statistical section the logic of the presentation, the figures/graphs should be revised.

Editorial guidance: The deconstruction of the 3- or 4-way ANOVA is also fine, although authors should clarify this further in the text. The authors should also ensure that the figures are presented in a manner that best reflects the statistical analyses.

Reply: We have now provided more rationale for the specific deconstruction of the ANOVAs in the text and we have worked hard to revise the figures to include panels depicting all significant main effects and interactions, which should facilitate navigation of the results.

3) The review from the first submission suggested to provide all the behavioral parameters analyzed during the marble-burying test for the 3 experiments to provide a more robust understanding of the role of sexual pheromones on affective behavior. For the experiment 2, the authors added the results of the distance travelled but report a lack of time to compute the time spent burying. We still believe that these results are necessary to the study and therefore should be included.

Editorial guidance: #3: We agree with reviewer 2 that the additional analyses should be included.

Reply: We have reviewed the video-recordings and included the data for the total time spent burying for Experiment 2. Please see the corresponding results section and Figure 3 panel B for graphical depictions of the results.

4) Moreover, for the experiment 3, the authors report a technical issue during the tests which did not allow to videotape the tests preventing to provide the latency to bury, the burying time and the locomotion. Technical issues are unfortunate, a replication of this experiment to collect all the data needed of the project would be welcome.

Editorial guidance: #4: We disagree with reviewer 2 that this experiment needs to be conducted again.

Reply: In accordance with the advice of the Editors, we have opted not to replicate Experiment 3.

5) The statistical analysis of the experiment 3 needs to be reviewed. Indeed, the 4 way ANOVA did not yield significant interaction. However, the authors report a strong trend for the Day x Initial exposition interaction and then follow up with a t-test analysis. Statistical analysis on trend effect are difficult to interpret and should be taken with caution. Moreover, it supports my comment above suggesting that this experiment should be remade to get a complete picture of the behaviors observed under this experimental conditions.

Editorial guidance: We agree in recommending more caution in the interpretation given that it relies on a statistical trend. This should also be acknowledged in the Discussion.

Reply: We have now acknowledged the statistical trend but justified the follow-up analysis by highlighting the large effect size associated with the interaction (see Page 10). We have also highlighted the p value for the interaction in Figure 4A”. There was no directed discussion of the results from Experiment 3 in any prior version of the report for us to revise and we found it difficult to insert a discussion of Experiment 3 outcomes given the focus and flow of the Discussion on interrelating the results from Experiments 1 and 2.

Minor comments :
1) The authors should read carefully the manuscript to remove some typos/comments through the manuscript. Line 7 page 9 the end of the sentence seems to be missing. Same comment line 44 page 9.
Reply: We apologize for the sloppiness in the revised manuscript. We have carefully proof-read this 2nd revision for typos and think we have caught all of the errors.

2) To clarify the experimental design in Figure 1, the authors could add the symbol male or female on the back of the mice as the sex do not appear in the figure.
Reply: We have inserted both the gender symbols and indicated that both adolescent and adult mice were tested in Figure 1. To minimize clutter, we only indicated these subject factors in the upper depictions of the marble-burying apparatus.

Round Three Decision Letter:-

It is a pleasure to accept your revised manuscript entitled "Modulation of marble-burying behavior in adult versus adolescent C57BL/6J mice of both sexes by ethologically relevant chemosensory stimuli" in its current form for publication in the Oxford Open Neuroscience. 

Please note that this journal operates with transparent peer review. This means that  the full peer review history of your article will publish online alongside your article. This includes reviewer comments, editor decision letters, and your author responses.

Next steps
You will receive an email from no-reply@scipris.com within roughly one week. This is your invitation to sign up for an account with SciPris, Oxford University Press’ author portal hosted by Aptara. You will need to create an account if you do not already hold one. Please register or log into your account and follow the online instructions which will guide you through signing your licence and paying the APC. The email and the portal have clearly signposted support options if you need any help during this process.

Please note that SciPris is a completely different system from ScholarOne, so your credentials to submit your manuscript here will not work there. Once you’ve created a SciPris account, you will be able to use it whenever you publish with Oxford Open Neuroscience or any OUP journal. Please note that OUP will only ever request payment for applicable fees be made via SciPris or to an OUP bank account. If you ever have concerns about the legitimacy of a request, please do not hesitate to contact a customer services agent via the SciPris portal or directly via oupsupport@scipris.com.

Thank you for your fine contribution.  On behalf of the Editors of the Oxford Open Neuroscience, we look forward to your continued contributions to the Journal.
